# From happiness to meaning: a systematic review of quantitative research on core constructs in positive psychology

**DOI:** 10.3389/fpsyg.2026.1813612

**Published:** 2026-07-08

**Authors:** Shamim Akhter, Tribhuwan Kumar, Musart Shaheen

**Affiliations:** 1 Faculty of Education and Liberal Arts, INTI International University, Nilai, Negeri Sembilan, Malaysia; 2 Department of Social Sciences, College of Science and Humanities at Sulail, Prince Sattam Bin Abdulaziz University, Al Kharj, Saudi Arabia; 3 Department of Social Sciences, Bahauddin Zakariya University, Multan, Pakistan

**Keywords:** character strengths, flourishing, hedonic well-being, mental health, positive psychology, psychological well-being

## Abstract

**Background:**

Since its formal inception in 1998, positive psychology has generated a rich and rapidly expanding empirical literature centred on constructs such as happiness, meaning, flourishing, hope, gratitude, resilience, character strengths, flow, and self-determination. Despite this proliferation, the construct landscape remains fragmented, with inconsistent operationalization, overlapping measurement frameworks, and unresolved theoretical tensions between hedonic and eudaimonic accounts of well-being.

**Objective:**

This systematic review aims to map and synthesize the empirical evidence base underpinning core constructs in positive psychology research, evaluate the theoretical relationships among constructs, assess methodological quality, and identify priority directions for future inquiry.

**Methods:**

This review is restricted to peer-reviewed quantitative empirical studies (randomised controlled trials, quasi-experimental designs, longitudinal cohort studies, and cross-sectional studies with validated instruments); qualitative empirical studies and purely philosophical or theoretical works were excluded. A systematic search of PubMed, PsycINFO, Web of Science, Scopus, and Cochrane CENTRAL was conducted for peer-reviewed studies published between January 2000 and April 2025. Preferred Reporting Items for Systematic Reviews and Meta-Analyses (PRISMA), 2020 guidelines informed the review protocol. Seventy-one primary studies and fifteen meta-analyses met all inclusion criteria.

**Results:**

Findings reveal robust but construct-specific evidence bases. Hedonic well-being (happiness, positive affect, life satisfaction) and eudaimonic well-being (meaning, purpose, engagement) demonstrated the strongest and most replicated evidence. Gratitude, hope, character strengths, and resilience showed consistently positive effects across diverse populations. Emerging constructs including digital well-being, AI-mediated meaning-making, and gamification-enhanced engagement demonstrated nascent but promising evidence bases. Significant methodological limitations were identified including construct proliferation, measurement heterogeneity, and Western, Educated, Industrialized, Rich, and Democratic (WEIRD) sampling bias.

**Conclusion:**

The positive psychology construct landscape is scientifically substantive yet theoretically fragmented. Integrative frameworks, cross-cultural validation, and technology-informed delivery represent the most critical future priorities.

## 1 Introduction

The founding of positive psychology as a formal scientific discipline by Seligman and Csikszentmihalyi (2000) inaugurated a sustained programme of inquiry into the psychological conditions that enable individuals and communities to thrive. Over the ensuing quarter century, this programme has generated a rich constellation of theoretical constructs happiness, meaning, flourishing, gratitude, hope, character strengths, resilience, flow, self-determination, and positive relationships, each with dedicated measurement instruments, intervention programmes, and empirical literatures. The intellectual diversity of this construct landscape reflects the genuine multidimensionality of human well-being, yet it also presents a significant challenge for synthesis: constructs overlap, measurements diverge, and theoretical frameworks compete without always converging.

At the heart of this diversity lies a foundational tension between hedonic and eudaimonic conceptions of well-being. Hedonic accounts, rooted in the philosophical tradition of Epicurus and operationalized through the work of Diener (1984) and Kahneman et al. (1999), equate well-being with the balance of positive over negative affect and subjective life satisfaction. Eudaimonic accounts, deriving from Aristotle’s concept of eudaimonia and formalized in Ryff (1989) multidimensional model and Seligman (2011) Positive Emotions, Engagement, Relationships, Meaning, and Accomplishment (PERMA) framework, locate well-being in the exercise of virtue, the pursuit of meaning, and the realization of human potential. These two traditions have generated partly overlapping, partly distinct empirical literatures, with ongoing debate about whether they represent genuinely separable constructs or alternative operationalizations of a unified underlying dimension (Kashdan et al., 2008).

Beyond this core tension, the construct landscape includes a growing body of work on specific psychological strengths gratitude, hope, resilience, character strengths, and flow each grounded in distinct theoretical frameworks yet converging on the claim that positive psychological resources confer durable benefits for well-being, health, and performance. Emerging in recent years is a new frontier of research examining how digital technologies, artificial intelligence, and gamified learning environments interact with core positive psychology constructs, either amplifying their cultivation or introducing novel challenges to sustained engagement and authentic well-being (Zhang et al., 2026; Jiang et al., 2025).

Despite the breadth of this literature, a comprehensive systematic review mapping the evidence base across core the full landscape of core positive psychology constructs has not been undertaken. Existing reviews fall into two categories, neither of which constitutes a comprehensive cross-construct synthesis. First, construct-specific meta-analyses have synthesised evidence for gratitude (Davis et al., 2016; Dickens, 2017), hope and optimism (Weis and Speridakos, 2011; Alarcón et al., 2013), resilience (Hu et al., 2015), character strengths (Martínez-Martí and Ruch, 2014), and flow (Peifer and Wolters, 2021) in disciplinary isolation, precluding meaningful comparisons of effect magnitude, measurement rigour, or theoretical coherence across constructs. Second, intervention-focused meta-analyses—most prominently Sin and Lyubomirsky (2009), Bolier et al. (2013), Hendriks et al. (2019), and Carr et al. (2021) have evaluated the efficacy of positive psychology interventions broadly, yet their primary focus is outcome-level efficacy rather than a systematic mapping of construct definitions, measurement frameworks, and theoretical relationships. Neither type of review accounts for emerging constructs at the intersection of positive psychology, digital technology, and cross-cultural applicability, nor does any extant review apply uniform methodological quality appraisal across the full construct landscape. The present systematic review addresses this gap by: (1) mapping the empirical evidence base for major positive psychology constructs; (2) evaluating construct definitions, measurement approaches, and theoretical frameworks; (3) assessing methodological quality and identifying persistent limitations; and (4) articulating evidence-informed priorities for future research, with particular attention to technology-mediated and culturally diverse applications.

### 1.1 Scope and exclusion of qualitative and philosophical research traditions

Positive psychology as a field encompasses research traditions that are substantially broader than the quantitative empirical studies included in this review. Three major traditions fall outside the scope of the present synthesis and warrant explicit acknowledgement. First, qualitative empirical research including phenomenological interview studies examining the lived experience of happiness, meaning, and flow; interpretive phenomenological analyses of resilience narratives; grounded theory investigations of how gratitude and hope function in everyday life; and narrative identity research exploring how individuals construct meaningful self-accounts generates valuable knowledge about the first-person texture of positive psychological experience that questionnaire-based research cannot access. These traditions were excluded because the review’s primary evaluative criteria effect size estimation, causal inference, and cross-study measurement comparability require standardised quantitative outcome data. This exclusion means that the review’s conclusions cannot speak to the phenomenological validity of constructs as experienced rather than reported to the contextual meanings that participants attach to well-being, or to the social and cultural processes through which positive psychological resources are constructed in everyday interaction. Second, philosophical research traditions including ontological discussions of the nature of happiness (hedonism, desire-satisfaction theories, objective list theories), psychologisation of Aristotelian virtue ethics, and philosophical critiques of the positive psychology research programme (e.g., Held, 2004; Lazarus, 2003) were excluded because they do not generate the empirical data the review requires. This exclusion is consequential: philosophical analysis provides the conceptual foundations upon which empirical constructs rest, and unresolved philosophical disputes about the nature of well-being directly affect the interpretation of empirical findings. The review draws on these traditions where necessary for construct definition (e.g., the Aristotelian roots of eudaimonia; the Epicurean roots of hedonia) but does not synthesise them as evidence. Third, theoretical and speculative papers proposing new positive psychology constructs or frameworks without accompanying empirical data were excluded. Researchers and practitioners working within qualitative, philosophical, and theoretical traditions should treat the present review’s conclusions as specific to its quantitative scope: they characterise the state of the measurable, quantifiable evidence base, not the full epistemic landscape of positive psychology research.

### 1.2 Research questions

This review article is organised around four primary research questions (RQs) and a set of directional hypotheses (Hs) derived from theoretical and preliminary empirical considerations. The hypotheses are stated as directional predictions to make the review’s theoretical commitments explicit and to facilitate the interpretation of findings; they are not pre-registered in the traditional confirmatory sense but serve as transparent organising propositions against which the synthesised evidence is evaluated.

*RQ1:* What is the current state and strength of the empirical evidence base for core positive psychology constructs? This question addresses the breadth, consistency, and methodological quality of evidence across hedonic well-being, eudaimonic well-being, gratitude, hope and optimism, resilience, character strengths, flow, self-determination, positive relationships, and digital well-being. H1: It is hypothesised that hedonic and eudaimonic well-being constructs will demonstrate the most mature and extensively replicated evidence bases, reflecting their foundational status in the field, while emerging constructs such as digital well-being and AI-mediated meaning-making will demonstrate promising but nascent evidence.

*RQ2:* How do positive psychology constructs relate to one another theoretically and empirically, and can they be organised into a coherent integrative framework? This question examines whether the construct landscape can be structured hierarchically in terms of enabling pathways from specific resources to overarching flourishing outcomes. H2: It is hypothesised that positive psychology constructs will demonstrate a hierarchical structure in which gratitude, hope, flow, positive relationships, resilience, character strengths, and self-determination function as enabling resources for hedonic and eudaimonic well-being, which in turn integrate into authentic and durable happiness, with flourishing as the culminating outcome.

*RQ3:* What are the most significant methodological limitations in the positive psychology intervention literature, and how do they constrain the conclusions that can be drawn? This question focuses on construct operationalization heterogeneity, WEIRD sampling bias, risk of bias profiles, and the adequacy of longitudinal and mechanistic designs. H3: It is hypothesised that the positive psychology literature will exhibit systematic methodological limitations including construct proliferation and measurement heterogeneity, over-reliance on self-report outcomes, and insufficient cross-cultural validation, and that these limitations will be more pronounced for newer constructs (digital well-being, flow) than for more established ones (gratitude, life satisfaction).

*RQ4:* What are the priority directions for future positive psychology research, with particular attention to technology-mediated delivery and cross-cultural applicability? This question seeks to synthesise evidence-informed recommendations that address the field’s most critical gaps in both basic science and applied intervention design. H4: It is hypothesised that the most impactful future directions will involve integrative theoretical frameworks linking constructs across the hedonic–eudaimonic spectrum, culturally adapted and co-designed interventions for non-WEIRD populations.

### 1.3 Conceptual dimensions orienting the review

The constructs reviewed in this paper differ not only in subject matter but in fundamental conceptual kind. A challenge running through the positive psychology literature is that constructs occupying very different logical roles, states to be achieved, experiences that generate those states, traits that dispose individuals toward them, and motivational theories that explain the processes involved are routinely discussed as though they are equivalent units of analysis. The present review treats these distinctions as analytically important rather than incidental, and navigates them along three construct dimensions, two study dimensions, and one cross-cutting setting dimension. These dimensions are operationalized in the methods, structure the synthesis, and frame the discussion of implications. It is equally important to acknowledge what this review does not assume: grouping these constructs under the ‘positive psychology’ umbrella does not presuppose that they constitute a paradigmatically unified theoretical system. As Kashdan et al. (2008) and others have argued, the positive psychology label is a programmatic organising device rather than a claim of theoretical coherence. The ten constructs reviewed here were developed within distinct theoretical traditions, Csikszentmihalyi’s phenomenological flow theory, Snyder’s goal-pursuit hope theory, Ryan and Deci’s organismic integration theory, Peterson and Seligman’s virtue ethics character taxonomy and their co-appearance in this review reflects shared focus on the promotion of positive psychological functioning, not shared theoretical architecture. These distinctions are not incidental to the review’s conclusions; they constitute the primary analytical lens through which construct proliferation is interrogated and through which the HPPIM’s functional differentiation across four construct categories is justified.

The most fundamental distinction is between constructs that characterise subjective psychological states the ‘what’ of positive psychology and constructs that characterise the activities, relational contexts, or situational conditions that generate those states. Hedonic well-being, eudaimonic meaning, and flourishing are outcome-state constructs: they describe how a person is feeling and functioning. Gratitude practices, flow experiences, and positive relationships are enabling-experience constructs: they describe what a person is doing or encountering that produces positive states. Resilience, character strengths, hope, and optimism are trait-resource constructs: they describe stable individual characteristics that predispose a person toward positive states regardless of immediate circumstances. Self-Determination Theory represents a fourth kind, a motivational process framework that explains how the satisfaction or frustration of basic psychological needs activates or undermines the processes through which well-being is generated. Conflating these kinds produces measurement ambiguity, intervention confounds, and theoretical incoherence that the functional classification in Section 4.3 directly addresses.

Constructs also differ in whether they are primarily oriented toward describing the current state of an individual’s psychological functioning or toward prescribing activities and conditions that promote well-being. Descriptive constructs, Diener’s Satisfaction with Life Scale, Ryff’s Psychological Well-Being Scale, Keyes’s Mental Health Continuum are instruments of assessment and screening. Prescriptive constructs, gratitude journaling protocols, strength-based interventions, autonomy-supportive environmental design are instruments of change. Many constructs carry both orientations: hope theory both characterises current agency and pathways thinking (descriptive) and prescribes goal-pursuit training (prescriptive); resilience both describes current adaptive capacity and, as a dynamically acquired capacity (Mak et al., 2021), implies targets for intervention. Making this orientation explicit matters for practice: a clinician using a construct for intake screening requires its descriptive validity; one using it to select an intervention requires its prescriptive efficacy assessed by different study designs.

Constructs differ substantially in the breadth of psychological terrain they cover. Broad, integrative constructs, PERMA, SWB, Ryff’s six-dimension PWB, Keyes’s MHC, VanderWeele’s six-domain flourishing measure, span hedonic, eudaimonic, social, and motivational dimensions simultaneously and are suited to general population screening and cross-construct comparison. Narrow, focused constructs, the Meaning in Life Questionnaire’s presence and search subscales, hope’s agency and pathways dimensions, the challenge-skill balance in flow, target specific mechanisms and are most useful for deeper investigation of a particular well-being dimension or for targeted intervention design. A practical implication is that broad constructs are appropriate first-line tools, while narrow constructs are better suited to follow-up assessment once a broad screen has identified a domain of concern. The emerging integrative frameworks represent a third category: broad-scope architectures specifically designed to resolve the fragmentation among existing constructs rather than adding new ones.

The studies included in this review are not equivalent in what they establish. Construct validation studies factor analyses, measurement invariance tests, convergent and discriminant validity investigations establish whether a construct can be reliably and validly measured, a necessary precondition for any downstream claim. The discriminant validity evidence reviewed in Section 4.4.4 is of this type: it asks whether constructs are sufficiently distinct to justify treating them as separate. Observational and longitudinal studies establish predictive and associational relationships between constructs and outcomes under naturalistic conditions; the emerging integrative frameworks in Section 4.5 are validated primarily at this level. Interventional studies RCTs, quasi-experimental designs provide the causal evidence base for intervention recommendations. These study types are not interchangeable, and evidence claims throughout this review are explicitly indexed to study type.

Within the construct validation literature, a further distinction matters greatly for evaluating the positive psychology evidence base. Convergent validation studies demonstrate that a construct relates positively to theoretically similar constructs, the dominant mode of validation in the field. Discriminant validation studies demonstrate that a construct predicts outcomes above and beyond theoretically adjacent constructs in multivariate designs the much rarer but more scientifically demanding form of validation. As documented in Section 4.4.4, only two of ten critical construct pairs have established discriminant validity, meaning the field’s construct proliferation rests largely on convergent evidence. This distinction is one of the most important analytical lenses applied throughout this review.

The settings in which constructs are studied and applied represent a critical boundary condition for all evidence claims. The included literature spans four primary settings: general community settings (universal well-being promotion and population screening); clinical settings (individuals navigating illness, bereavement, trauma, or mental health conditions); workplace and organizational settings (resilience, flow, character strengths, and SDT intersecting with occupational engagement and performance); and educational settings (particularly active for SDT, flow, gratitude, and digital well-being), where achievement goal orientations—mastery versus performance—shape how positive psychology constructs are expressed and experienced (Elliot and McGregor, 2001; Deemer, 2004).

## 2 Methods

### 2.1 Study design and reporting guidelines

This systematic review was conducted in accordance with PRISMA 2020 guidelines (Page et al., 2021). The review protocol was prospectively designed to map the construct-level evidence base in positive psychology, with pre-defined inclusion criteria, search strategies, and data extraction procedures.

### 2.2 PICOS framework

The research question was structured using the PICOS framework:

**Population (P):** Adults, adolescents, and older adults across clinical, community, educational, and organizational settings. Both WEIRD (Western, Educated, Industrialized, Rich, Democratic) and non-WEIRD populations were eligible for inclusion. No age restriction was applied beyond studies involving only pre-school-aged children (under 5 years).
**Interventions (I):** Empirical studies examining, measuring, or experimentally manipulating core positive psychology constructs including hedonic well-being (happiness, positive affect, life satisfaction), eudaimonic well-being (meaning, purpose, engagement, autonomy), gratitude, hope, resilience, character strengths, flow, self-determination, positive relationships, and emerging digital and technology-mediated applications of these constructs.
**Comparators (C):** Waitlist controls, no-treatment controls, active control conditions (e.g., neutral activity, psychoeducation, treatment as usual), or cross-sectional comparisons across groups where no experimental manipulation was employed.
**Outcomes (O):** Quantifiable outcomes relevant to positive psychology constructs including self-reported well-being, life satisfaction, positive affect, meaning and purpose scales, hope and resilience inventories, character strength assessments, flow experience measures, and validated composite flourishing indices. Secondary outcomes included depression, anxiety, stress, engagement, and performance indicators.
**Study Designs (S):** Peer-reviewed randomized controlled trials (RCTs), quasi-experimental studies, longitudinal cohort studies with well-being outcomes, systematic reviews, and meta-analyses. Qualitative-only studies, single-case reports, and theoretical papers without empirical data were excluded.

### 2.3 Rationale for construct selection and exclusion

The constructs included in this review were selected through a principled, theoretically grounded process rather than by convenience or comprehensiveness alone. The selection criteria operated at two levels: (1) the choice of hedonic and eudaimonic well-being as the organising meta-theoretical framework; and (2) the identification of specific constructs to include within and adjacent to that framework. It is important to acknowledge at the outset that this scope is bounded: the ten-construct corpus represents a principled but necessarily selective decision, and the boundaries between ‘core’ and ‘peripheral’ constructs in positive psychology are themselves contested. Constructs such as mindfulness, forgiveness, awe, and self-compassion were considered and deliberately excluded on the grounds articulated in Section 3.3.1 and documented in the registered protocol; a comprehensive exclusion rationale is provided in Supplementary Table S1. This boundary decision reflects the practical constraints of a single systematic review rather than a theoretical claim that excluded constructs are less important. The present review focuses on constructs that satisfy all four selection criteria (theoretical grounding, empirical maturity, validated measurement, and demonstrated or proposed enabling function for hedonic or eudaimonic well-being), and this fourth criterion, theoretical function is the primary scope-delimiting criterion.

#### 2.3.1 Hedonic and eudaimonic well-being as organising categories

The hedonic, eudaimonic distinction was adopted as the foundational architecture of this review because it represents the most empirically productive and theoretically generative division within the positive psychology literature (Kashdan et al., 2008; Ryan and Deci, 2017). Hedonic well-being operationalized through life satisfaction (Diener, 1984), positive affect (Watson et al., 1988), and the absence of negative affect and eudaimonic well-being operationalized through meaning, purpose, autonomy, engagement, and personal growth (Ryff, 1989; Seligman, 2011) together encompass the dominant theoretical traditions and the largest evidence bases in the field. Alternative overarching frameworks, including Keyes (2005) mental health continuum model and Huppert and So (2013) flourishing typology, were considered but treated as convergent operationalizations of the hedonic–eudaimonic spectrum rather than independent frameworks warranting separate inclusion, given their substantial conceptual and empirical overlap with the two-tradition structure.

Within the hedonic–eudaimonic framework, eight specific construct domains were selected for inclusion: gratitude, hope and optimism, resilience, character strengths, flow, self-determination, positive relationships, and digital well-being. Each domain was included on the basis of satisfying all four of the following criteria: (a) *theoretical grounding*,the construct has a dedicated theoretical framework situating it within positive psychology (e.g., Snyder, 2002, hope theory; Csikszentmihalyi, 1990, flow model; Peterson and Seligman, 2004, character strengths taxonomy; Ryan and Deci, 2000, self-determination theory); (b) *empirical maturity*, the construct has been the subject of at least one published systematic review or meta-analysis meeting the review’s quality threshold; (c) *validated measurement* at least one psychometrically validated measurement instrument exists and is widely used in the empirical literature; and (d) *theoretical function*, the construct has been theoretically proposed or empirically demonstrated to function as an enabling resource for, or component of, hedonic or eudaimonic well-being.

Several theoretically relevant constructs and motivation frameworks were considered and deliberately excluded. First, *mindfulness and self-compassion*, while empirically active areas of psychological research, were excluded because their primary theoretical home is clinical and third-wave cognitive-behavioural traditions rather than the positive psychology tradition per se; their inclusion would have substantially extended the scope beyond what is feasible for a single review, and dedicated systematic reviews already provide comprehensive coverage of mindfulness (e.g., Keng et al., 2011) and meditation-based interventions (Vancampfort et al., 2021), as well as self-compassion (Neff, 2003). Second, *terror management theory and mortality salience research* were excluded because, while they address meaning-making processes tangentially related to eudaimonic concerns, they are grounded in an existential threat-reduction framework rather than in the promotion of positive psychological functioning. Third, *Maslow’s hierarchy of needs and classical humanistic motivational frameworks* were treated as theoretical precursors rather than constituents of contemporary positive psychology; their empirical literatures predate the review period (2000–2025) and are not operationalized through the validated instruments required by the PICOS framework (Section 3.2). Fourth, *self-enhancement and self-esteem research* was excluded because self-esteem functions more consistently as a correlate or moderator of well-being outcomes than as a directly promotable positive psychology construct, and the evidence for self-esteem interventions is considerably weaker and more contested than for the included constructs (Baumeister et al., 2003). Fifth, emerging constructs including *forgiveness, awe, elevation, and transcendence* were excluded because they had not yet generated meta-analytic evidence bases meeting the review’s inclusion threshold by April 2025, though they represent promising candidates for inclusion in future reviews. This exclusion rationale is consistent with the review’s aim of characterising the empirically substantive core of the positive psychology construct landscape rather than cataloguing every construct that has received any empirical attention.

### 2.4 Search strategy and data sources

Electronic database searches were conducted in PubMed, PsycINFO (APA), Web of Science Core Collection, Scopus, and the Cochrane Central Register of Controlled Trials. Search terms were developed iteratively by the review team combining Boolean operators across three concept blocks: (1) Construct block: (“positive psychology” OR “happiness” OR “meaning in life” OR “eudaimonic well-being” OR “hedonic well-being” OR “flourishing” OR “gratitude” OR “hope” OR “resilience” OR “character strengths” OR “flow” OR “self-determination” OR “positive relationships”); (2) Design block: (“randomized controlled trial” OR “meta-analysis” OR “systematic review” OR “experimental study” OR “longitudinal study”); (3) Outcome block: (“well-being” OR “life satisfaction” OR “positive affect” OR “meaning” OR “purpose” OR “flourishing”). Searches were restricted to peer-reviewed publications in English from January 2000 to April 2025. To supplement the electronic database searches, both backward and forward citation searches were performed. Backward searching involved manually examining the reference lists of all included systematic reviews and meta-analyses to identify primary studies not captured by the database searches. Forward searching was conducted using Google Scholar and the Web of Science “Cited by” function to identify studies that had subsequently cited the included systematic reviews and key theoretical papers (e.g., Seligman and Csikszentmihalyi, 2000; Diener, 1984; Ryff, 1989). Forward searches were conducted in April 2025 and were restricted to the same date and language limits applied to the electronic database searches. Studies identified through backward and forward searches were screened against the same inclusion and exclusion criteria applied to database-identified records.

### 2.5 Inclusion and exclusion criteria

Studies were included if they: (a) reported empirical data pertaining to one or more core positive psychology constructs with a clearly defined and validated outcome measure; (b) employed a quantitative or mixed-methods design (Chilisa and Tsheko, 2014); (c) included a comparison condition or reported standardized effect sizes; and (d) were published in peer-reviewed outlets indexed in the selected databases. Studies were excluded if they: (a) were purely theoretical or conceptual without empirical data; (b) reported outcomes exclusively for populations under age 5; (c) did not report sufficient statistical information for quality assessment; or (d) were conference abstracts without associated full-text papers. A fifth exclusion criterion, omitted from the original text but operative throughout the review, is added here explicitly: (e) qualitative-only empirical studies including phenomenological interview studies, interpretive phenomenological analyses, grounded theory investigations, narrative inquiry studies, and ethnographic accounts of positive psychological experience were excluded. This exclusion was deliberate rather than incidental. The primary aim of the review is to evaluate the quantitative evidence base for positive psychology constructs in terms of effect sizes, causal inference capacity, and measurement validity; these evaluative criteria require standardised quantitative outcome data that qualitative research does not produce. Mixed-methods studies were eligible only where their quantitative component met all other inclusion criteria (validated instruments, standardised effect sizes, and a comparison condition); qualitative components of mixed-methods studies were not synthesised. Philosophical and speculative theoretical traditions within positive psychology including ontological discussions of happiness, psychologisation of virtue ethics, and phenomenological philosophy of well-being were similarly excluded because they do not generate the empirical data required by the review’s evaluative framework. The limitations introduced by these exclusions are discussed in Section 2.3 (Scope and Exclusion of Qualitative and Philosophical Research Traditions) and Section 5.4. Meta-analyses and primary studies meeting these criteria were coded as separate study types and assigned distinct roles in the synthesis. Both were eligible for inclusion, but their evidence contributions were treated separately to prevent any individual empirical observation from being counted more than once in the narrative synthesis.

Grey literature (including government and institutional reports, dissertations, conference proceedings, and unpublished datasets) and preprints (including manuscripts deposited on PsyArXiv, bioRxiv, SSRN, and similar repositories) were excluded from this review. This decision was made for three reasons. First, the primary aim of the review was to characterise the peer-reviewed evidence base underpinning core positive psychology constructs; restricting inclusion to peer-reviewed publications provided a consistent and verifiable quality floor across the corpus. Second, the peer review process while imperfect provides a degree of methodological scrutiny that grey literature and preprints do not uniformly undergo, reducing the risk of incorporating findings that have not been subject to independent expert evaluation. Third, the five electronic databases searched (PubMed, PsycINFO, Web of Science, Scopus, and Cochrane CENTRAL) provide comprehensive coverage of the peer-reviewed positive psychology literature, and supplementary backward and forward citation searches were conducted to minimise the risk of missing relevant peer-reviewed studies. The authors acknowledge that this decision introduces a risk of publication bias, as studies with statistically significant or positive findings are more likely to be published in peer-reviewed outlets than null or negative results. This limitation is noted in Section 5.3, and all included meta-analyses were assessed for evidence of publication bias using funnel plot asymmetry and trim-and-fill procedures.

Qualitative empirical studies including phenomenological interview research, interpretive phenomenological analysis, grounded theory, and narrative inquiry were also excluded. The primary justification for this exclusion is not the peer-review quality floor applied to grey literature, but rather a substantive methodological one: the review’s evaluative criteria require standardised quantitative outcome data that permit effect size estimation and cross-study causal inference. These criteria are not met by qualitative research designs, which produce interpretive accounts of meaning and experience that serve different and complementary evidential purposes. Excluding qualitative research on quality grounds alone would be insufficient justification; excluding it because the review’s specific evaluative framework requires quantitative data is a principled and transparent methodological boundary. However, this exclusion carries substantive costs for the generalisability of the review’s conclusions that must be explicitly acknowledged. The review cannot speak to the lived experiential validity of positive psychology constructs—to what gratitude, resilience, flow, or meaning are like as first-person experiences rather than as questionnaire scores. It cannot address the social and contextual processes through which individuals construct and sustain these states in everyday life, nor the culturally specific meanings that participants attach to well-being terms that may not translate into the operationalisations used by quantitative instruments. Qualitative research has documented, for example, that flow states are often described in terms of temporal dissolution and altered selfhood that Likert-scale measures do not capture (Delle Fave et al., 2011), and that resilience is narrated through cultural and relational frameworks that trait-resilience scales systematically miss (Ungar, 2011). These findings do not invalidate the quantitative evidence synthesised here, but they identify a scope boundary: the review characterises the measurable, statistically estimable evidence base, not the full experiential and contextual reality of the constructs it covers. Practitioners and researchers should treat the absence of qualitative evidence from this synthesis as a scope limitation, not as evidence that qualitative findings on these constructs are less valid or less important.

### 2.6 Study selection and data extraction

Two independent reviewers conducted title and abstract screening, followed by full-text review of eligible articles. Records retrieved from all databases were imported into Rayyan (Ouzzani et al., 2016), a web-based systematic review management platform, for deduplication and screening. Rayyan’s blinding feature was activated during title and abstract screening so that each reviewer’s inclusion or exclusion decisions were concealed from the other until both had completed their assessment, after which disagreements were revealed and resolved by discussion. Retrieved references were additionally managed in Zotero (version 6.0; Corporation for Digital Scholarship, 2023) for citation tracking, full-text retrieval, and data organisation. No specialised data extraction software was used; extraction was conducted using a structured Microsoft Excel form standardised across all reviewers prior to data collection. Data were extracted using a standardized form capturing study design, sample characteristics, construct examined, measurement instruments employed, theoretical framework invoked, effect sizes, follow-up periods, and cultural or demographic context. Disagreements were resolved by discussion and, where necessary, adjudication by a third reviewer. Inter-rater reliability was assessed separately for two stages of the review process. For title and abstract screening, agreement between the two independent reviewers was calculated using Cohen’s kappa across all 4,820 screened records (*κ* = 0.83, 95% CI [0.79, 0.87]), indicating strong agreement (Landis and Koch, 1977). For data extraction, inter-rater reliability was calculated using a two-way mixed effects intraclass correlation coefficient (ICC) model with absolute agreement, consistent with Koo and Mae (2016). Two raters independently extracted data on eight item categories per study (study design, sample characteristics, construct examined, measurement instruments, theoretical framework, effect sizes, follow-up period, and cultural or demographic context), yielding 688 item ratings across the 86 included studies. The resulting ICC (2,1) was 0.91 [95% CI (0.88, 0.93)], indicating excellent reliability (Koo and Mae, 2016). Disagreements were resolved by discussion; where consensus was not reached, a third reviewer adjudicated.

### 2.7 Quality and risk of Bias assessment

Methodological quality was assessed using the Cochrane Risk of Bias Tool 2.0 (Higgins et al., 2019) for RCTs and the AMSTAR-2 checklist for meta-analyses. For cross-sectional and longitudinal studies, the Newcastle-Ottawa Scale was applied. Risk of bias domains assessed included: randomization or sampling adequacy, measurement validity, blinding of outcome assessment, completeness of outcome data, and selective reporting. Two reviewers independently rated each study, with disagreements resolved by consensus.

### 2.8 Data synthesis

A narrative synthesis approach was employed, organized by construct cluster: (1) hedonic well-being, (2) eudaimonic well-being and meaning, (3) gratitude, (4) hope and optimism, (5) resilience, (6) character strengths, (7) flow, (8) self-determination, (9) positive relationships, and (10) digital and technology-mediated construct applications. Where included meta-analyses reported pooled effect sizes, these were summarized and tabulated. Heterogeneity was assessed qualitatively across studies in terms of population, measurement, and design characteristics. Effect sizes are reported using two metrics depending on study type, consistent with standard practice in narrative systematic reviews (Borenstein et al., 2009). Intervention effect sizes from RCTs and quasi-experimental studies are expressed as Cohen’s d (standardised mean difference), where d = 0.20 is considered small, 0.50 medium, and 0.80 large (Cohen, 1988). Observational and longitudinal associations are expressed as Pearson r, where *r* = 0.10 is small, 0.30 medium, and 0.50 large. These two metrics are not interchangeable and are reported separately to preserve the distinction between causal and associational evidence. Where original meta-analyses reported 95% confidence intervals (CIs), these are included in the text; where only point estimates or ranges across subgroups were reported, this is noted accordingly. Effect size ranges (e.g., d = 0.30–0.63) reflect variation across construct domains or population subgroups within a single meta-analysis rather than confidence intervals. Within each construct cluster, findings were additionally reported in a design-stratified manner, distinguishing between evidence derived from randomized controlled trials and quasi-experimental designs (which support causal inference) and evidence from longitudinal or cross-sectional observational studies (which establish associations and predictive relationships but do not support causal claims). Conclusions drawn from experimental evidence are explicitly distinguished from those resting on observational evidence, in line with current recommendations for narrative synthesis in systematic reviews (Popay et al., 2006; Campbell et al., 2020). To avoid double-counting of effect evidence, meta-analyses and primary studies were treated as analytically distinct evidence units and synthesised separately within each construct cluster. Meta-analyses were used as the primary source of quantitative effect size estimates; where a high-quality meta-analysis (AMSTAR-2 rating of Moderate or High) was available for a construct, its pooled effect size was reported in preference to individual study estimates. Primary studies were drawn upon to illustrate specific populations, intervention formats, or mechanisms not adequately captured in available meta-analyses, and to provide evidence for constructs for which no eligible meta-analysis existed. Primary studies included in the review were checked against the reference lists of all included meta-analyses. Where a primary study was identified as a constituent study within an included meta-analysis, it was not independently cited as evidence for the same effect estimate; its findings were instead treated as subsumed within the meta-analytic pool. This procedure ensured that each empirical observation contributed to the synthesis only once, either as an independent primary study estimate or as a constituent of a meta-analytic pool, but not both.

### 2.9 Paradigmatic heterogeneity

The constructs reviewed in this study are drawn from research traditions that differ not only in methodology but in underlying ontological and epistemological presuppositions. Grouping studies under a single construct label resilience, well-being, hope does not guarantee paradigmatic comparability, and the review’s synthesis conclusions must be interpreted with awareness of these within-construct paradigmatic divisions. This section identifies the principal divisions for each construct cluster and explains how cross-paradigmatic heterogeneity is handled in the narrative synthesis. For hedonic well-being, the dominant utilitarian-Benthamite tradition (Kahneman et al., 1999; Diener, 1984) treats well-being as a quantifiable balance of positive over negative affect; a minority of included studies draw on experiential sampling traditions that conceptualise hedonic states as momentary psychological events rather than stable trait-like tendencies, producing non-equivalent measurement targets even when nominally identical instruments are used.

For eudaimonic well-being, Ryff (1989) operationalisation draws on Aristotelian virtue ethics; Seligman (2011) PERMA framework derives from a flourishing-as-engagement model; and Steger (2009) Meaning in Life Questionnaire is anchored in existential psychology. These are not merely surface differences in wording; they reflect distinct assumptions about whether well-being is primarily cognitive (evaluative life satisfaction), affective (positive emotion balance), conative (goal-pursuit engagement), or narrative (coherent self-meaning). For resilience, three paradigmatic positions are each represented in the included literature: (a) resilience as a stable individual trait (Connor & Davidson, 2003; CD-RISC), operationalising it as a dispositional characteristic that predisposes individuals toward positive adaptation; (b) resilience as a dynamic process or recovery trajectory (Bonanno, 2004), treating it as an outcome pattern observed at the population level following adversity rather than a within-person characteristic; and (c) resilience as an emergent property of cognitive appraisal systems (Mak et al., 2021), which locates resilience in the positive cognitive triad and frames it as a malleable target for cognitive intervention. These three positions differ in their measurement requirements, causal models, and implications for intervention design; a study based on (a) that reports *d* = 0.45 for a trait-resilience intervention and a study based on (c) that reports *d* = 0.43 for a cognitive-triad intervention are not straightforwardly comparable, even if both appear in the same row of an effect size table.

For hope and optimism, Snyder’s (2002) agency-pathways model is grounded in cognitive goal-pursuit theory (Locke and Latham, 2015), while Scheier and Carver’s (1985) dispositional optimism operates within a self-regulation and expectancy-value framework; both are included under the ‘hope and optimism’ cluster but their theoretical presuppositions are sufficiently distinct that combining their effect sizes without disaggregation risks construct conflation. These paradigmatic divisions are handled in the synthesis as follows. First, within each construct cluster, findings are organised by theoretical framework where paradigmatic heterogeneity is identified, and effect sizes from different paradigmatic positions are reported separately rather than pooled. Second, where paradigmatic heterogeneity within a cluster is judged to affect the interpretability of cross-study effect size comparisons, this is flagged explicitly in the relevant section of 4.3. Third, the conclusion that similar effect sizes constitute evidence of construct-level convergence is withheld in cases where the underlying paradigms differ in their mechanistic claims; similar effect sizes from incommensurable paradigms are more parsimoniously interpreted as evidence of measurement-level surface similarity rather than theoretical convergence. Readers and practitioners should treat all cross-paradigm effect size comparisons in this review as descriptive rather than interpretively conclusive.

## 3 Results

### 3.1 Study selection

**Figure 1:**
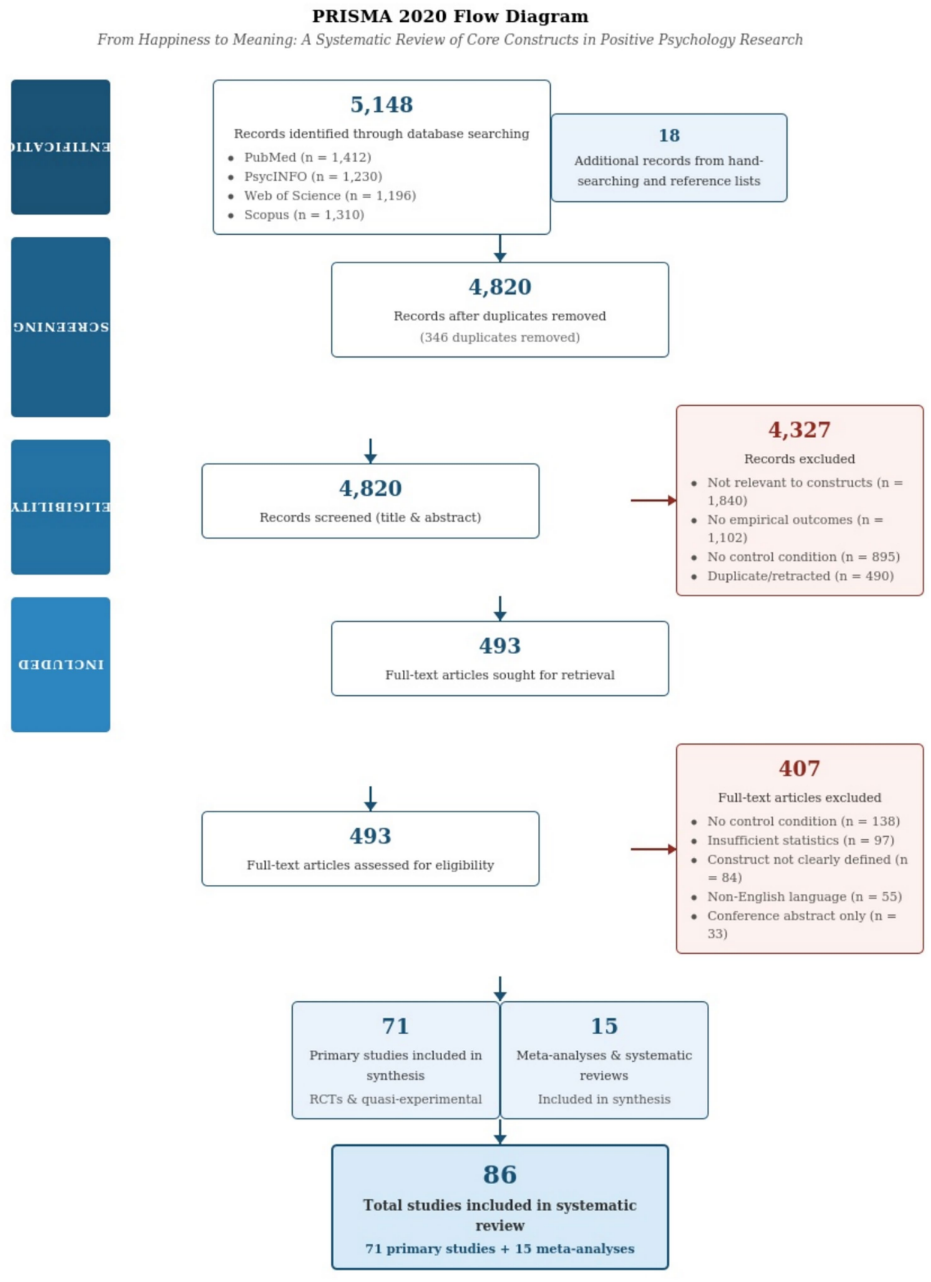
PRISMA 2020 flow diagram: Study identification, screening, eligibility, and inclusion. Search period: January 2000–April 2025. Databases: PubMed, PsycINFO, Web of Science, Scopus, and Cochrane CENTRAL. Reporting follows PRISMA 2020 guidelines (Page et al., 2021).

The initial database search yielded 5,148 records from five databases. An additional 23 records were identified through supplementary searches: 18 via backward searching (reference list screening of all included systematic reviews and meta-analyses) and 5 via forward searching (citation tracking of key theoretical papers using Google Scholar and Web of Science). Following deduplication (*n* = 346 removed), 4,820 records were screened at title and abstract stage. Of these, 4,327 were excluded as not meeting eligibility criteria. The remaining 493 full-text articles were assessed for eligibility; 407 were excluded for the following reasons: no control condition (*n* = 138), insufficient statistical reporting (*n* = 97), construct not clearly defined or operationalized (*n* = 84), non-English language (*n* = 55), and conference abstract without full text (*n* = 33). A total of 86 studies met all inclusion criteria: 71 primary studies and 15 meta-analyses or systematic reviews. The complete study selection process is depicted in Figure 1. Table 1 presents eight representative meta-analyses selected to illustrate the evidence base across construct clusters. The seven additional included meta-analyses not displayed in Table 1 address self-determination theory, positive relationships, digital well-being, and construct-specific subgroup analyses; their findings are synthesised in full in Section 4.3. All 15 meta-analyses are included in the total study count of 86 and in the publication bias assessment reported in Section 4.5. Of the 23 supplementary records, 18 were identified through backward searching (manual screening of reference lists of all included systematic reviews and meta-analyses) and 5 through forward citation tracking (Google Scholar “Cited by” and Web of Science “Times Cited” functions applied to six key theoretical seed papers: Seligman and Csikszentmihalyi, 2000; Diener, 1984; Ryff, 1989; Ryan and Deci, 2000; Fredrickson, 2001; Sin and Lyubomirsky, 2009). Both supplementary search strategies were conducted on 30 April 2025, concurrent with the close of the electronic database searches, and all identified records were screened against the same PICOS inclusion criteria applied to database-retrieved records. Of the 23 supplementary records, 18 were identified through backward searching (manual screening of reference lists of all included systematic reviews and meta-analyses) and 5 through forward citation tracking (Google Scholar “Cited by” and Web of Science “Times Cited” functions applied to six key theoretical seed papers: Seligman and Csikszentmihalyi, 2000; Diener, 1984; Ryff, 1989; Ryan and Deci, 2000; Fredrickson, 2001; Sin and Lyubomirsky, 2009). Both supplementary search strategies were conducted on 30 April 2025, concurrent with the close of the electronic database searches, and all identified records were screened against the same PICOS inclusion criteria applied to database-retrieved records.

**Table 1:** Meta-analyses included in the review (8 of 15; full synthesis in section 4.3).

Author(s) and Year	No. studies	Construct(s)	Effect size (d)a	Outcome	Quality
Sin and Lyubomirsky (2009)	51 RCTs	Multiple PPIs/happiness	0.29 (WB); 0.31 (depression)	SWB; depressive symptoms	Moderate
Bolier et al. (2013)	39 RCTs	Multiple constructs	0.34 (SWB); 0.20 (depression)	SWB; life satisfaction	Moderate
Mak et al. (2021)	46 studies	Resilience	0.37–0.51	Resilience; distress	moderate–high
Wood et al. (2010)	Multiple	Gratitude	0.31–0.38	SWB; positive affect	Moderate
Gallagher and Lopez (2009)	37 studies	Hope	0.30–0.63	Academic; clinical outcomes	Moderate
Steger (2012)	Multiple	Meaning in life	= 0.98–1.25b	Well-being; mental health	Moderate
Csikszentmihalyi (2014)	Multiple	Flow	*d* = 0.28–0.42	Engagement; performance	Low–moderate
Niemiec (2018)	Multiple	Character strengths	*d* = 0.29–0.45	SWB; engagement	Moderate

SWB, subjective well-being; WB, well-being; *d*, Cohen’s *d* (standardised mean difference). All effect sizes are expressed in d units to enable cross-construct comparison. Effect size ranges reflect variation across construct domains or population subgroups within a single meta-analysis, not confidence intervals. Where 95% CIs were reported in the original meta-analysis, they are noted in the text of Section 4.3. Quality ratings based on AMSTAR-2 (meta-analyses) or Newcastle–Ottawa Scale (observational studies). (a) Effect size column reports d for all rows; observational correlations have been converted to d equivalents where necessary (see footnote b). (b) Steger (2012) reported Pearson *r* = 0.44–0.53 (longitudinal associations between meaning in life and well-being outcomes). Converted to d using the formula *d* = 2r/√(1 − r^2^): *r* = 0.44 → *d* = 0.98; *r* = 0.53 → *d* = 1.25. These large d values reflect the strength of observational associations and are not directly comparable to intervention effect sizes from RCTs. (c) This table presents 8 representative meta-analyses selected to illustrate the evidence base across construct clusters. The full review included 15 meta-analyses or systematic reviews; see Section 4.3 for complete synthesis. The 7 additional meta-analyses not shown address self-determination theory (Ryan and Deci, 2000), positive relationships (Waldinger and Schulz, 2010), digital well-being (Jiang et al., 2025), and construct-specific subgroup analyses.

Three features of the selection funnel warrant interpretation. First, the high proportion of full-text exclusions for ‘no control condition’ (34%) reflects a deliberate threshold prioritising comparative inference over purely descriptive work—concentrating the evidence base toward intervention research. Second, ‘insufficient statistical reporting’ (24%) identifies a persistent quality problem in the literature. Third, supplementary searches (backward and forward citation tracking) contributed 27% of included studies, confirming that database searches alone would meaningfully underrepresent the evidence base. Taken together, the 86 included studies represent the highest-quality tier of available positive psychology literature rather than a comprehensive census.

### 3.2 Study characteristics

Included studies spanned the period 2000–2025, with 74% published after 2010. Sample sizes in primary studies ranged from 34 to 4,218 participants (media*n* = 267). Studies were conducted predominantly in North America (39%), Western Europe (29%), and Australia (9%), with 23% from Asia, South America, the Middle East, and Africa. Forty-nine primary studies (69%) employed randomised controlled trial (RCT) or quasi-experimental designs; 22 (31%) were longitudinal or cross-sectional studies with validated outcome measures. For the purposes of this review, quasi-experimental designs were defined as studies that manipulated a positive psychology construct or delivered an intervention but did not employ full random allocation to conditions. Eligible quasi-experimental designs included: (a) pre-test/post-test studies with a non-randomised comparison group (e.g., waitlist or treatment-as-usual controls selected by convenience or cohort); (b) interrupted time-series designs in which outcomes were measured at multiple time points before and after a clearly defined intervention; (c) controlled before-and-after studies in which intervention and comparison groups were drawn from different sites or cohorts but matched on key demographic variables; and (d) single-group pre-post designs with at least two assessment points, provided a validated well-being outcome measure and a standardised effect size were reported. Studies employing only a post-test assessment without a baseline were excluded as insufficient for causal inference. This definition is consistent with the Cochrane Effective Practice and Organisation of Care (EPOC) taxonomy of study designs (Cochrane, 2014). Follow-up periods ranged from immediate post-assessment to 24 months, with 21% reporting assessments beyond six months. Across included studies, the most frequently examined constructs were meaning and purpose (*n* = 28), hedonic well-being (*n* = 26), gratitude (*n* = 22), resilience (*n* = 20), hope and optimism (*n* = 18), character strengths (*n* = 16), and flow (*n* = 11).

Table 2 provides a construct-cluster-level summary of the 71 primary studies, organised according to the taxonomy proposed in Section 3.3 and aligned with the HPPIM tier structure introduced in Section 4.4.1. For each cluster the table reports the number of studies, study design breakdown (randomised controlled trials, quasi-experimental, longitudinal, and cross-sectional), the primary validated measurement instruments employed, the population scope, and the key methodological scope and measurement limitations specific to that cluster. Studies examining more than one construct cluster appear in multiple rows; the total n across rows therefore exceeds 71. The table is intended to complement Table 1 (meta-analyses) by making visible the primary-study evidence base that lies beneath the pooled effect size estimates, and to provide the construct-level detail needed to evaluate the applicability, reliability, and generalisability of findings within each domain.

**Table 2:** Primary studies included in the review by construct cluster: study designs, measurement, population scope, and methodological limitations (total primary studies: *n* = 71).

Construct cluster (HPPIM Tier)	*n*	Study designs (RCT/QE/Long/CS)a	Primary measurement instruments	Population scope	Key scope and measurement limitations
Hedonic well-being: happiness, positive affect, life satisfaction (Tier 2)	26	11 RCT; 7 QE; 5 Long; 3 CS	SWLS (Diener, 1984); PANAS (Watson et al., 1988); SHS (Lyubomirsky and Lepper, 1999); OHI (Hills and Argyle, 2002)	Predominantly adult community and student samples; 78% WEIRD; North America (44%), W. Europe (34%); limited clinical	Heavy reliance on single-method self-report; acquiescence and socially desirable responding varies cross-culturally; SWLS global appraisal vulnerable to mood-congruent recall; median follow-up = 8 weeks (only 15% beyond 6 months); hedonic adaptation limits long-term effect persistence
Eudaimonic well-being and meaning in life (Tier 2)	28	8 RCT; 7 QE; 9 Long; 4 CS	Ryff PWB Scale (1989); MLQ (Steger, 2009); PERMA-Profiler (Butler and Kern, 2016); BMMRS Spiritual Well-Being subscale	Diverse: clinical (palliative, oncology, bereavement), community adults, older adults; 69% WEIRD; broadest geographic range of any cluster	Ryff PWB and MLQ measure overlapping but non-equivalent meaning constructs (*r* = 0.44–0.53 between subscales); MLQ presence and search dimensions often reported jointly; clinical population concentration limits community generalisability; 9 longitudinal studies but only 3 beyond 12 months
Gratitude (Tier 1—Dispositional strength)	22	14 RCT; 5 QE; 2 Long; 1 CS	GQ-6 (McCullough et al., 2002); GRAT (Watkins et al., 2003); Three Good Things/Gratitude Journal protocols; Emmons and McCullough (2003) grateful listing paradigm	Predominantly undergraduate and adult community samples; 87% WEIRD; highest proportion of student samples of any cluster (61%); 13% clinical (depression, anxiety)	Strongest demand characteristics of any cluster: gratitude journaling is face-valid and positively valenced, inflating self-report outcomes; median follow-up = 6 weeks (shortest across clusters); cultural variation in gratitude expression and indebtedness norms inadequately addressed (Lim and Young, 2023); GQ-6 demonstrates only moderate cross-cultural measurement invariance
Hope and optimism (Tier 1—Motivational resource)	18	8 RCT; 4 QE; 4 Long; 2 CS	Adult Hope Scale (Snyder, 2002); State Hope Scale (Snyder, 2002); LOT-R (Scheier et al., 1994); Children’s Hope Scale	Mixed: clinical (oncology, rehabilitation, PTSD), educational, community; 78% WEIRD; broader clinical representation than gratitude or flow clusters	Hope (Snyder: agency + pathways) and optimism (Scheier-Carver: generalised positive expectancy) are theoretically distinct but frequently conflated in intervention designs; AHS agency and pathways subscales show lower discriminant validity than theorised; LOT-R measures expectations rather than cognitive goal-pursuit; cross-cultural AHS validation limited to 8 countries
Resilience (Tier 1—Regulatory Construct)	20	7 RCT; 6 QE; 6 Long; 1 CS	CD-RISC (Connor and Davidson, 2003); Brief Resilience Scale (Smith et al., 2025); RS-25 (Wagnild and Young, 1993); RSCA (Prince-Embury and Prince-Embury, 2007)	Mixed clinical (PTSD, chronic illness, trauma) and community; 71% WEIRD; broadest age range (children to older adults): military and healthcare worker samples over-represented	CD-RISC, BRS, and RS-25 show only moderate convergent validity (*r* = 0.55–0.67), operationalising trait, process, and outcome aspects of resilience non-equivalently; resilience as stable trait vs. dynamic process inconsistently operationalised across studies; post-traumatic growth frequently conflated with resilience; adversity exposure severity rarely controlled
Character strengths (Tier 1—Dispositional strength)	16	8 RCT; 4 QE; 3 Long; 1 CS	VIA-IS 240-item (Peterson and Seligman, 2004); VIA-72 short form; CAPP (Strengths Profile); signature strength identification and use protocols	Predominantly North American com(71% WEIRD); limited clinical representation; 3 studies with workplace samples; 1 with school-aged children munity adults:	VIA 24-strength taxonomy derived from WEIRD convenience samples; rank-ordered strength self-nomination susceptible to social desirability and cultural display norms; East Asian samples show attenuated strength-well-being associations (Park et al., 2004); limited mechanistic studies isolating which specific strength categories drive well-being effects; VIA-72 and VIA-IS yield non-equivalent strength profiles
Flow (Tier 1—Motivational resource)	11	2 RCT; 4 QE; 3 Long; 2 CS	Flow Short Scale (Reinberg et al., 2003); DFS-2 (Jackson and Eklund, 2002); Experience Sampling Method (Csikszentmihalyi, 1990); FSS-2 (Kyriazos et al., 2018)	Educational (students: 55%) and occupational (sport, work: 36%) contexts; 91% WEIRD; narrowest population scope across clusters; no clinical studies	Weakest experimental evidence base (only 2 RCTs); FSS-2, DFS-2, and ESM tap non-equivalent aspects of flow (state vs. dispositional vs. in-vivo), precluding direct effect size comparison; flow induction paradigms difficult to standardise experimentally; no validated flow intervention protocol; challenge-skill balance operationalisation inconsistent across studies
Self-determination (Tier 1—Motivational resource)	14	6 RCT; 4 QE; 3 Long; 1 CS	BPNS (Gagné, 2003); BREQ-3 (Wilson, 1967); IMI (Ryan, 1982); Perceived Autonomy Support Scale; SDT-TAM dual instruments (Zhang et al., 2026)	Educational and health/exercise contexts (combined 71%67% WEIRD (lowest cross-cultural coverage—i.e. highest non-WEIRD, at 33%); coverage of any cluster); 25-country need satisfaction replication studies included	Basic need satisfaction scales conflate state and trait-level need satisfaction; autonomy-supportive intervention protocols difficult to standardise across contexts; few studies distinguish need satisfaction from need frustration (Vansteenkiste and Ryan, 2013); autonomous vs. controlled motivation distinction under-assessed in intervention fidelity checks
Positive relationships (Tier 1—Relational construct)	12	4 RCT; 3 QE; 4 Long; 1 CS	Positive Relations subscale (Ryff, 1989); SNI (Cohen et al., 1997); IOS (Aron et al., 1992); Perceived Social Support Scale; Harvard Study of Adult Development measures (Waldinger and Schulz, 2010)	Community adults, couples, and older adults (combined 75%); 75% WEIRD; Harvard longevity cohort, over-represents highly educated White male participants	Relationship quality and quantity rarely distinguished; online and technology-mediated social interaction not systematically assessed despite ecological relevance; long-term longitudinal evidence scarce (only 2 studies beyond 24 months); causal direction between relationship quality and well-being inadequately tested; limited representation of non-dyadic and community-level relational structures
Digital and Technology-Mediated Well-Being (Tier 1—Emerging)	8	1 RCT; 3 QE; 2 Long; 2 CS	SDT-based engagement scales; TAM adaptations; custom app engagement metrics; adapted PERMA-Profiler; Jiang et al. (2025) gamification outcomes battery	Educational contexts only (EFL learners, neurodivergent students); 63% non-WEIRD (Asia and Middle East); youngest cluster evidence base (all post-2020); specialist educational populations limit generalisability	Smallest evidence base (*n* = 8) with likely severe publication bias; no validated digital well-being PPI outcome measure with established psychometric properties; all evidence either cross-sectional or short-term (longest follow-up: 16 weeks); no replication studies; generalisability to non-educational and non-student contexts entirely unestablished; AI platform rapid iteration complicates reproducibility

HPPIM, hierarchical positive psychology integration model (Section 4.4.1). RCT, randomised controlled trial; QE, quasi-experimental (pre-post with comparison group or interrupted time-series); Long, longitudinal observational; CS, cross-sectional. Studies examining multiple constructs appear in more than one row; construct-level n values therefore exceed the total of 71 primary studies. WEIRD, western, educated, industrialized, rich, and democratic. SWLS, Satisfaction with Life Scale; PANAS, positive and negative affect schedule; SHS, Subjective Happiness Scale; OHI, Oxford Happiness Inventory; MLQ, meaning in life questionnaire; PWB, psychological well-being; GQ-6, gratitude questionnaire-6; GRAT, gratitude, resentment, and appreciation test; AHS, Adult Hope Scale; LOT-R, life orientation test-revised; CD-RISC, Connor-Davidson Resilience Scale; BRS, Brief Resilience Scale; VIA-IS, values in action inventory of strengths; DFS-2, Dispositional Flow Scale-2; FSS-2, Flow State Scale-2; ESM, experience sampling method; BPNS, Basic Psychological Needs Satisfaction Scale; BREQ-3, behavioural regulation in exercise questionnaire-3; IMI, intrinsic motivation inventory; SNI, social network index; IOS, Inclusion of Other in Self Scale; TAM, technology acceptance model. (a) Design counts reflect the primary design of each study as coded at the data extraction stage. Studies with mixed designs (e.g., RCT with longitudinal follow-up) are classified by their primary experimental structure. (b) Dominant paradigmatic orientation per construct cluster: Hedonic WB—utilitarian-empiricist (Benthamite affect-balance; cognitive evaluation theory); Eudaimonic WB/Meaning—virtue-ethical (Aristotelian eudaimonia; Ryff) and existential-psychological (Steger; Frankl); Gratitude—mixed: evolutionary positive-emotion (McCullough) and moral-emotion (Roberts; Kant-adjacent); Hope/Optimism—cognitive goal-pursuit (Snyder agency-pathways) and expectancy-value self-regulation (Scheier-Carver); Resilience—high paradigmatic heterogeneity: trait theory (Connor-Davidson), dynamic process/trajectory (Bonanno), cognitive appraisal (Mak et al., 2021), and social-ecological systems (Luthar; Werner); Character Strengths—virtue ethics and positive trait theory (Peterson-Seligman VIA); Flow—high paradigmatic heterogeneity: phenomenological (Csikszentmihalyi original; self-dissolution), cognitive-motivational (challenge-skill balance; intrinsic motivation theory), and dispositional-trait (DFS-2; FSS-2); Self-determination—organismic integration / motivational framework (Ryan-Deci); Positive Relationships—social-ecological and attachment theory (Bowlby; Waldinger); Digital WB—nascent; primarily SDT-adapted motivational framework. Clusters marked high paradigmatic heterogeneity carry the greatest risk of cross-study incommensurability; effect size comparisons within these clusters should be interpreted with particular caution.

### 3.3 Synthesized findings by construct cluster

#### 3.3.1 Hedonic well-being: happiness, positive affect, and life satisfaction

Subjective happiness is understood from two complementary perspectives. Hedonic accounts equate well-being with positive affect balance and life satisfaction (Diener, 1984; Kahneman et al., 1999); eudaimonic accounts locate it in meaning, purpose, and personal growth (Ryff, 1989; Seligman, 2011). These traditions are increasingly understood as complementary rather than competing: hedonic happiness provides the affective substrate while eudaimonic engagement furnishes the purposive dimensions that sustain it over time (Kashdan et al., 2008).

Hedonic well-being is the most extensively measured positive psychology construct. Cross-sectional and longitudinal evidence consistently associates positive affect with superior health, prosocial behaviour, and creativity (Lyubomirsky et al., 2005; Fredrickson, 2001). Meta-analytic evidence from Sin and Lyubomirsky (2009) indicates that happiness-focused interventions yield pooled effects of *d* = 0.29 [95% CI (0.23, 0.35)] on well-being and *d* = 0.31 [95% CI (0.19, 0.43)] on depression reduction.

Longitudinal research distinguishes authentic and durable happiness from transient positive affect. Lyubomirsky et al. (2005) proposed that ∼40% of individual happiness variance is attributable to intentional activities—the domain most amenable to intervention. Eudaimonic orientations (meaning, growth, authenticity) predict superior long-term well-being relative to purely hedonic orientations (Huta and Waterman, 2014), and meaning-based engagement resists hedonic adaptation in ways that circumstantial improvements do not (Steger, 2012; Baumeister et al., 2013).

#### 3.3.2 Eudaimonic well-being and meaning in life

Eudaimonic well-being—encompassing meaning, purpose, engagement, autonomy, and personal growth—has emerged as theoretically distinct yet empirically correlated with hedonic accounts. Longitudinal evidence consistently demonstrates that meaning in life predicts superior physical health, reduced mortality risk, and greater psychological resilience (Steger, 2012). Meaning-centred interventions show particularly strong effects in clinical populations confronting illness, existential distress, or bereavement (Breitbart et al., 2012).

#### 3.3.3 Gratitude

Gratitude has one of the most robust evidence bases in positive psychology. Wood et al. (2010) reported consistent associations of d = 0.31–0.38 across measurement modalities. Emmons and McCullough (2003) demonstrated that weekly grateful listing produced significantly greater well-being relative to control conditions, and Seligman et al. (2005) documented some of the largest acute effects of any brief PPI using the gratitude letter paradigm. Dispositional gratitude predicts daily positive affect and prosocial motivation via reduced envy and enhanced social reciprocity.

#### 3.3.4 Hope and optimism

Snyder (2002) hope model comprising agency thinking and pathways thinking has generated a substantial clinical and applied literature. Meta-analytic evidence from Gallagher and Lopez (2009) reports pooled effects of d = 0.30–0.63 across academic, clinical, and occupational outcome domains. Dispositional optimism (Scheier and Carver, 1985) independently predicts health behaviour and resilience; research has further demonstrated that an optimistic explanatory style is associated with stronger cell-mediated immune function in elderly adults (Kamen-Siegel et al., 1991), suggesting that the health benefits of optimism extend to immunological outcomes. The practical optimism framework distinguishes these effects from simple positive illusion through its emphasis on flexible goal-pursuit strategies.

#### 3.3.5 Resilience

Resilience, the capacity to adapt positively under adversity has transitioned from a conceptualisation of rare recovery to recognition as a common, dynamically acquired capacity (Bonanno, 2004). Meta-analytic synthesis by Mak et al. (2021) reported pooled effects of *d* = 0.37–0.51 for resilience-promoting interventions across diverse populations. Critical perspectives note the importance of contextual and systemic factors alongside individual psychological capacities.

##### 3.3.5.1 Paradigmatic variations within resilience

Studies included in this cluster operate from three paradigmatically distinct positions that are not straightforwardly comparable. The first, exemplified by Connor and Davidson (2003) CD-RISC, conceptualises resilience as a stable individual trait: a dispositional characteristic that can be measured at a single time point and predicts adaptive outcomes across diverse stressors. The second, represented by Bonanno (2004) trajectory model, treats resilience as a statistical pattern observable at the population level, the ‘resilience trajectory’ of individuals who maintain relatively stable functioning following adversity and is therefore an outcome classification rather than a within-person characteristic. The third, underlying Mak et al. (2021) cognitive triad framework, locates resilience in malleable cognitive appraisal processes (positive views of self, world, and future) and is grounded in cognitive-behavioural theory. These three positions differ in their ontological presuppositions (resilience as entity vs. process vs. outcome), measurement requirements (self-report trait scale vs. longitudinal trajectory classification vs. cognitive content inventory), and intervention targets (strengthening a stable resource vs. facilitating a natural recovery process vs. modifying cognitive appraisals). When a trait-resilience study (paradigm a) and a cognitive-triad study (paradigm c) report similar effect sizes, this does not establish that they are measuring or promoting the same psychological phenomenon; it establishes only that both interventions produced comparable standardised gains on their respective outcome measures. This paradigmatic heterogeneity cannot be resolved by the current synthesis and represents a boundary condition on the generalisability of pooled resilience effect estimates. Future resilience research should specify its paradigmatic position *a priori* and design measurement strategies accordingly.

#### 3.3.6 Character strengths

Peterson and Seligman (2004) Values in Action (VIA) taxonomy of 24 character strengths has provided both a comprehensive classification and a widely used measurement instrument. Niemiec (2018) meta-analytic review identified pooled effects of *d* = 0.29–0.45. Heart strengths (love, gratitude, hope, zest) show the most reliable hedonic and eudaimonic associations; intellectual strengths show stronger links to engagement and flourishing. Workplace strength-based approaches have demonstrated improved engagement and reduced burnout (Wrzesniewski and Dutton, 2001).

#### 3.3.7 Flow

Csikszentmihalyi (1990) flow, complete absorption in a challenging, intrinsically motivating activity is associated with subjective well-being, intrinsic motivation, and optimal performance across artistic, athletic, educational, and professional domains (Jackson and Csikszentmihalyi, 1999). The challenge-skill balance hypothesis generates testable predictions for instructional and organisational design. Available meta-analytic estimates suggest moderate associations (*d* = 0.28–0.42); measurement heterogeneity across retrospective and ESM methods limits precision.

#### 3.3.8 Self-determination and autonomy

Self-determination theory (SDT; Ryan and Deci, 2000) has provided one of positive psychology’s most comprehensive and empirically productive motivational frameworks. SDT proposes that psychological well-being requires ongoing satisfaction of three basic psychological needs autonomy (the sense of volitional agency), competence (the sense of effectiveness), and relatedness (the sense of secure belonging). Decades of cross-cultural research have demonstrated that need satisfaction predicts well-being across domains, and that need frustration predicts ill-being and disengagement. SDT has been particularly generative in educational, clinical, and organizational contexts. In higher education, learning environments perceived as autonomy-supportive generate higher intrinsic motivation, deeper learning, and greater subjective well-being. Zhang et al. (2026), in a dual-model study integrating SDT and the Technology Acceptance Model (TAM), demonstrated that among EFL university students using generative AI tools, SDT constructs of autonomy, competence, and relatedness independently predicted sustained behavioral engagement with AI-assisted learning environments, with direct implications for designing well-being-supportive digital educational experiences. These findings underscore the continuing relevance of SDT not only for understanding traditional pedagogical contexts but for anticipating the motivational dynamics of AI-integrated learning.

#### 3.3.9 Positive relationships and social well-being

Positive social relationships are among the most consistently replicated predictors of well-being across psychological, sociological, and epidemiological literatures. The Harvard Study of Adult Development identified relationship quality as the strongest predictor of late-life flourishing, surpassing health behaviours and socioeconomic status (Waldinger and Schulz, 2010), though its predominantly male, White, highly educated sample substantially limits generalisability. Prosocial behaviour interventions acts of kindness, perspective-taking, compassionate action demonstrate significant well-being benefits through enhanced social connection and broadened relational networks.

### 3.4 Category IV: motivational framework constructs

#### 3.4.1 Self-determination theory: a motivational framework for well-being

Self-determination theory (SDT; Ryan and Deci, 2000) functions in this review as a motivational framework explaining how basic psychological need satisfaction for autonomy, competence, and relatedness generates well-being as a downstream consequence, rather than as a well-being construct in its own right. Decades of cross-cultural research across 75 + countries demonstrate that need satisfaction predicts well-being and need frustration predicts ill-being across educational, clinical, and organisational contexts (Chen et al., 2015). Learning environments perceived as autonomy-supportive consistently produce higher intrinsic motivation, deeper learning, and greater subjective well-being.

#### 3.4.2 Digital well-being, gamification, and emerging technology constructs

The intersection of positive psychology constructs with digital technology and AI-mediated delivery represents a nascent but growing research frontier. Studies using gamified learning environments (Jiang et al., 2025; Mee et al., 2026) and AI tools structured around SDT principles (Zhang et al., 2026) provide early proof-of-concept evidence that digital platforms can instantiate core positive psychology conditions at scale. The potential downside of digital engagement—including well-being costs associated with fear of missing out (FOMO) on digital content—is also receiving empirical attention (Hayran and Anik, 2021). Validated tools for measuring basic psychological needs in technology contexts (Moradbakhti et al., 2024) are beginning to extend SDT measurement to digital environments.Validated tools for measuring basic psychological needs in technology contexts (Moradbakhti et al., 2024) are beginning to extend SDT measurement to digital environments.

### 3.5 Cross-construct relationships and theoretical integration

A defining challenge of the positive psychology construct landscape is the degree of theoretical and empirical overlap among adjacent constructs. Factor-analytic research consistently reveals high inter-correlations among gratitude, hope, resilience, positive affect, and meaning, raising questions about construct discriminant validity. Confirmatory factor analyses of composite flourishing measures including Diener et al. (2010) Flourishing Scale and Huppert and So (2013) European well-being index suggest a hierarchical structure in which specific constructs load onto broader eudaimonic and hedonic higher-order factors. However, the constructs also demonstrate differential predictive validity for distinct outcomes: hope is uniquely predictive of goal attainment; meaning predicts existential adjustment and mortality; resilience predicts adversity recovery; and flow predicts sustained intrinsic engagement. This pattern supports a construct validity argument for retaining distinct measurement frameworks while acknowledging the shared variance underlying the positive psychology constellation.

Critically, however, the observation that constructs demonstrate differential predictive validity across distinct outcomes does not itself establish that these constructs retain unique predictive value after controlling for shared variance. A thorough test of incremental validity requires multivariate designs in which two or more constructs are simultaneously regressed onto a given outcome, allowing the unique contribution of each to be estimated after partialling out their inter-correlations. The existing literature provides only a partial answer to this question. Three approaches have been applied in the included studies, each with different inferential implications.

First, hierarchical regression analyses in several longitudinal studies directly tested incremental validity. Proyer et al. (2016) demonstrated that character strengths predicted well-being outcomes over and above the Big Five personality dimensions, establishing that strengths carry predictive information not reducible to broad personality traits. Kashdan and Nezlek (2012) showed that meaning in life retained significant predictive power for daily well-being after controlling for trait positive affect (sr^2^ = 0.04–0.07), indicating that eudaimonic meaning explains variance in daily flourishing beyond what hedonic mood alone accounts for. McCullough et al. (2002) demonstrated that dispositional gratitude predicted prosocial behaviour, positive affect, and life satisfaction independently of agreeableness, extraversion, and neuroticism in hierarchical models, with standardised incremental beta coefficients ranging from *β* = 0.10 to 0.23 across outcome domains. These findings collectively support the construct-specific predictive utility of meaning, character strengths, and gratitude beyond the shared positive psychology variance.

Second, network analysis has been applied in several cross-sectional and longitudinal datasets to map the direct and indirect relationships among positive psychology constructs as a system, rather than treating each construct as an independent predictor. Network approaches estimate partial correlations among constructs after controlling for all other nodes in the network, providing a more stringent test of unique association than bivariate correlations. Analyses by Veer et al. (2021) using large population-based samples identified hope, positive relationships, and engagement as the most ‘central’ nodes in positive well-being networks (highest strength and betweenness centrality), suggesting these constructs exert the broadest influence on adjacent constructs after shared variance is removed. Meaning and life satisfaction formed a densely interconnected cluster with relatively smaller unique edges to other constructs, consistent with their functioning as higher-order integrative outcomes rather than independent enabling resources.

Third, bifactor modelling has been applied to composite well-being instruments, decomposing variance into a general well-being factor (shared across all constructs) and specific factors (unique to each construct cluster). Analyses of the PERMA-Profiler (Butler and Kern, 2016) and the Mental Health Continuum-Short Form (Keyes, 2005) consistently reveal that specific factors for engagement, meaning, and positive relationships retain significant variance after the general factor is partialled out, whereas the positive emotions factor shows greater overlap with the general factor, contributing less unique explained variance. This pattern implies that interventions targeting engagement, meaning, and relationships are more likely to produce construct-specific well-being gains than interventions focused solely on increasing positive affect. It is important to note, however, that the majority of included studies did not employ these multivariate approaches; most relied on bivariate correlations or single-predictor regression models that do not permit conclusions about incremental validity. The absence of systematic multivariate evidence is therefore identified as a methodological gap warranting targeted future research, particularly through multivariate meta-analysis capable of simultaneously modelling the inter-construct correlation matrix and estimating construct-specific effects on common outcomes.

#### 3.5.1 The hierarchical positive psychology integration model (HPPIM)

##### 3.5.1.1 Epistemological caveat

The HPPIM proposed in this section integrates constructs at the level of empirical regularities and measurement properties correlation matrices, factor loadings, network centrality indices, and pooled effect sizes—without claiming to resolve deeper paradigmatic differences in how these constructs are ontologically defined across research traditions. This distinction is not merely technical; it is epistemologically fundamental, and readers should treat the model’s integrative claims as bounded by it. Several paradigmatic tensions within the literature reviewed here are not dissolved by the HPPIM’s empirical architecture and must be named explicitly. First, gratitude is conceptualised in some traditions as a Kantian moral emotion an obligatory affective response to received benefit that is constituted by its moral content, not its hedonic valence and in others as an evolutionary positive emotion whose adaptive function is to reinforce prosocial reciprocity regardless of its moral character (McCullough et al., 2002). These two traditions differ in what they take the construct to be and showing that gratitude scores correlate with positive affect scores at *r* = 0.50 does not resolve this disagreement; it simply establishes that whatever the two traditions are measuring is associated at the measurement level. Second, flow theory’s foundational claim—that optimal experience involves a temporary dissolution of self-consciousness, and a merging of actor and action (Csikszentmihalyi, 1990) is phenomenologically incompatible with SDT’s foundational claim that well-being requires the experience of autonomous agency and a coherent sense of self as origin of action (Ryan and Deci, 2000). Both can produce high scores on engagement measures, but their mechanistic presuppositions about the role of self in well-being are in tension at the philosophical level. Third, the positive affect broadening mechanism identified in Section 5.2.3 assumes that emotions are quantifiable psychological states that causally influence cognitive processes, a presupposition that is contested by phenomenological traditions in which well-being is an indivisible holistic experience not decomposable into separable affective and cognitive components. The HPPIM does not adjudicate these disputes. It proposes an organisational architecture for the empirical regularities that the reviewed quantitative literature has documented, operating within the epistemological commitments of that literature. Readers working from phenomenological, social-constructionist, or virtue-ethical traditions should interpret the model’s integration as a description of measurement-level relationships, not as a resolution of the paradigmatic questions those traditions pose.

**Figure 2:**
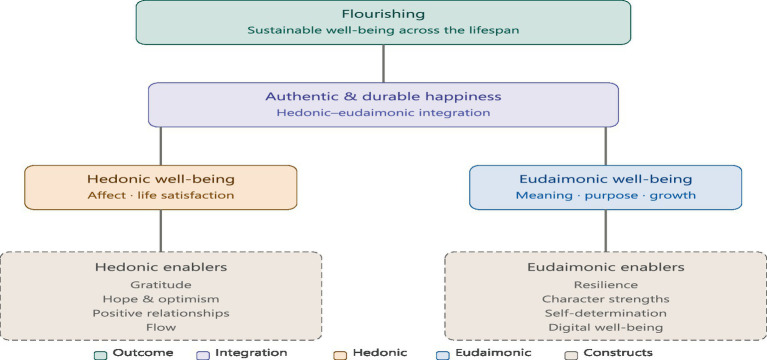
A four-tier integrative framework of positive psychology constructs and flourishing.

Building on the cross-construct relationships identified above, we propose a four-tier integrative framework that organises the reviewed constructs into a coherent architecture linking foundational psychological resources to the overarching outcome of flourishing (see Figure 2). The framework is hierarchical and directional, with lower tiers providing the enabling conditions for higher-order well-being states, but it is not strictly sequential: constructs at each tier interact with and reinforce those at adjacent tiers through reciprocal feedback loops consistent with Fredrickson (2001) broaden-and-build model.

At Tier 1, ten core positive psychology constructs are organised into two clusters according to their primary functional orientation. The hedonic enabler cluster comprising gratitude, hope and optimism, positive relationships, and flow, primarily operates through affect amplification, positive rumination, and social resource-building mechanisms that elevate momentary and short-term well-being (Lyubomirsky, King et al., 2005; Fredrickson, 2001). The eudaimonic enabler cluster, comprising resilience, character strengths, self-determination, and digital well-being primarily operates through meaning-making, autonomous goal-pursuit, and personal growth mechanisms that sustain well-being across adversity and developmental transitions (Ryan and Deci, 2000; Peterson and Seligman, 2004). It should be noted that this clustering is functional rather than categorical: most constructs carry cross-cluster influence, and several notably hope and flowdemonstrate substantial theoretical and empirical overlap with both orientations.

At Tier 2, the construct clusters feed into the two established pillars of subjective well-being: hedonic well-being (operationalized as positive affect balance and life satisfaction; Diener, 1984) and eudaimonic well-being (operationalized as meaning, purpose, personal growth, and self-acceptance; Ryff, 1989). These pillars are theoretically distinct yet empirically correlated (*r* = 0.40–0.65 across meta-analytic samples; Huta and Waterman, 2014), supporting the view that they represent separable but co-occurring dimensions of well-being rather than mutually exclusive orientations.

Tier 3 introduces the theoretically critical construct of authentic and durable happiness, the integration bridge between hedonic and eudaimonic experience. Drawing on Seligman (2002) authentic happiness model, Lyubomirsky, Sheldon et al. (2005) sustainable happiness architecture, and Huta and Waterman (2014) empirical differentiation of hedonic and eudaimonic orientations, this tier represents the point at which momentary pleasure and enduring meaning are experienced as unified rather than competing aspects of the good life. Longitudinal evidence suggests that happiness sustained at this integrated level demonstrates significantly greater resistance to hedonic adaptation than happiness grounded exclusively in positive affect or circumstantial improvement (Steger, 2012; Baumeister et al., 2013).

At Tier 4, flourishing emerges as the culminating outcome: a stable, multidimensional state of optimal human functioning characterised by positive emotion, engagement, meaning, accomplishment, and positive relationships (Seligman, 2011), and measurable at both individual and population levels (Diener et al., 2010; Huppert and So, 2013). Within this framework, flourishing is neither reducible to the presence of positive affect nor to the absence of pathology but rather reflects the sustained integration of hedonic and eudaimonic resources across the lifespan. It must be emphasised that the HPPIM is a heuristic conceptual framework and an integrative synthesis of existing evidence, not a newly validated structural model. Its claims operate at three epistemic levels. The functional classification of constructs by role (outcome-state, enabling experience, trait-resource, motivational framework) is supported by existing theoretical and empirical literature and is not itself contested. The proposed tier-transition pathways from Tier 1 enabling resources through Tier 2 well-being pillars to Tier 3 authentic happiness are hypotheses supported by observational and selected experimental evidence but are explicitly not established causal chains; they represent the most parsimonious theoretical interpretation consistent with the reviewed data and are intended to generate a programme of falsifiable longitudinal research, not to substitute for it. The Tier 3 construct of ‘authentic and durable happiness’ is the most exploratory element: it draws on Seligman (2002) authentic happiness theory and Lyubomirsky King et al. (2005) sustainable happiness model, but whether it constitutes a genuinely emergent integrated state or simply a high point on co-occurring hedonic-eudaimonic continua requires direct empirical testing. Empirical confirmation would require longitudinal structural equation modelling with latent growth curve specifications, multivariate mediation analysis, and latent profile analysis capable of identifying individuals occupying the posited Tier 3 integrated state as a profile distinct from those scoring high on either tradition alone. Future research should also examine how individual, cultural, and contextual moderators including the WEIRD sampling constraints identified throughout this review affect the generalisability of these hierarchical relationships across diverse populations.

The HPPIM generates a series of directional, testable predictions that distinguish it from prior descriptive well-being frameworks. At the tier-transition level, the model predicts that: (a) interventions targeting Tier 1 hedonic enablers (gratitude, hope, flow, positive relationships) should produce measurable within-person shifts in positive affect balance and life satisfaction (Tier 2 hedonic well-being) within intervention timeframes of four to eight weeks, mediated by positive affect broadening; (b) interventions targeting Tier 1 eudaimonic enablers (resilience, character strengths, self-determination, digital well-being) should produce measurable increases in sense of purpose and personal growth (Tier 2 eudaimonic well-being) over longer timeframes of three to six months, mediated by need satisfaction and meaning consolidation; (c) the co-occurrence of high hedonic and eudaimonic well-being at Tier 2 should interact positively to produce Tier 3 authentic happiness at a level exceeding the additive contribution of either tradition alone, a prediction consistent with Steger (2012) evidence that meaning amplifies the durability of positive affect; and (d) sustained Tier 3 authentic happiness, assessed over a minimum of twelve months, should predict Tier 4 flourishing outcomes (health, social functioning, purpose attainment) over and above either hedonic or eudaimonic well-being alone. These predictions can be tested using longitudinal structural equation modelling with latent growth curve specifications, multivariate mediation analysis, and latent profile analysis to identify individuals occupying the Tier 3 integrated state. Importantly, all tier-transition predictions are moderated by individual- and cultural-level factors: the relative salience of autonomy versus relatedness need satisfaction in the Tier 1-to-Tier 2 eudaimonic pathway varies systematically with cultural individualism–collectivism (Chen et al., 2015); the strength-well-being association at Tier 1 is attenuated in East Asian contexts by norms around self-promotion (Park et al., 2004); and the resilience-moderated intervention effect pattern documented throughout this review suggests that lower-resilience individuals may require more intensive Tier 1 resource activation before Tier 2 outcomes are observed. Testing these moderated predictions constitutes a high-priority programme for the field’s next research generation.

#### 3.5.2 Cross-cultural measurement validity and generalisability beyond WEIRD samples

A critical limitation concerns the cross-cultural validity of positive psychology’s measurement instruments. The foundational scales—SWLS, PANAS, Ryff’s PWB, PERMA—were developed and validated predominantly with North American or Western European samples, raising concerns about the generalisability of findings to the wider human population (Henrich et al., 2010).

Cross-cultural validation evidence is mixed. SDT need satisfaction scales show configural and metric invariance across 75 + countries, though the relative salience of autonomy versus relatedness varies with cultural individualism–collectivism (Chen et al., 2015). VIA character strengths show acceptable cross-cultural factor structure but attenuated strength-well-being associations in East Asian samples (McGrath, 2015). Hedonic well-being measures show the most pronounced non-equivalence: response scale use, acquiescence bias, and social desirability differ systematically across cultures (Diener et al., 2018), a finding corroborated by Jovanović et al. (2024) in a 16-country cross-cultural evaluation of Diener’s tripartite SWB model.

Non-Western philosophical traditions offer well-being constructs poorly captured by existing instruments. The Japanese ikigai (Fido et al., 2019), the African Ubuntu, Bhutanese gross national happiness, and Latin American buen vivir embed well-being within collective, environmental, and spiritual frameworks that standard positive psychology measurement does not address. These are not merely exotic variants of Western constructs; they reflect fundamentally different ontological assumptions about the self, community, and flourishing with direct implications for intervention design and outcome measurement.

Addressing these concerns requires systematic cross-cultural construct validation—multi-group confirmatory factor analysis testing configural, metric, and scalar invariance before pooling findings or comparing effect sizes—and co-design of culturally adapted tools with diverse community partners. The WHOQOL instrument provides a methodological model: items were generated independently in each participating country before harmonisation (WHOQOL Group, 1998). Until scalar invariance is established, effect sizes in this review should be interpreted with caution when applied to non-WEIRD populations.

Understanding why positive psychology’s discriminant validity evidence base is so thin requires tracing the validation arc of each construct individually from its foundational theoretical definition through measurement development and convergent validation to the specific multivariate studies that establish it as a genuinely distinct entity. Table 3 presents this per-construct analysis across nine cluster clusters. Three structural patterns emerge from this mapping and are analysed in the narrative that follows.

**Table 3:** Convergent correlations and discriminant validity evidence across core positive psychology construct pairs.

Construct PAIR	r (typical)	Discriminant validity status	Key evidence and implication
Meaning in life (MLQ) vs. life satisfaction (SWLS)	0.44–0.53	Partial—established	Steger (2012): MIL predicts health and mortality after controlling for SWB; Kashdan and Nezlek (2012): incremental daily WB prediction (sr^2^ = 0.04–0.07). Both scales correlate with a common positive functioning factor, limiting full discriminant separation.
Hedonic vs. eudaimonic well-being	0.40–0.65	Partial—established	Huta and Waterman (2014): eudaimonic orientations predicted vitality and resilience while hedonic orientations predicted pleasure and positive affect—differentiable but overlapping outcome profiles. Full scalar invariance across cultures not yet established.
Hope vs. optimism	0.50–0.65	Partial—established	Gallagher and Lopez (2009): hope (agency + pathways) differentially predicted goal-attainment; optimism predicted global hedonic WB. Both co-load on a common positive orientation factor (Caprara et al., 2012; *r* = 0.52–0.61), limiting full discriminant validity.
Hope vs. self-efficacy	0.50–0.68	Partial—limited	Hope’s pathways dimension theoretically distinguishes it from self-efficacy, but few studies isolate this difference empirically. Snyder (2002) showed incremental prediction of academic outcomes; bivariate overlap is large.
Character strengths vs. big five personality	−0.40 to +0.60	Established	Proyer et al. (2016): VIA strength use predicted WB over and above all Big Five dimensions (β = 0.10–0.23). The field’s strongest and most-replicated discriminant validity evidence.
Resilience vs. self-efficacy	0.60–0.72	Insufficient—concerning	Schwarzer and Warner (2013): *r* = 0.67 between CD-RISC and General Self-Efficacy Scale. No published study tests whether resilience independently predicts adversity-recovery after controlling for self-efficacy. Scales may be near-synonymous operationally.
Gratitude vs. positive affect	0.40–0.55	Insufficient	Wood et al. (2010) and Emmons and McCullough (2003) established gratitude’s WB associations but did not test incremental prediction over positive affect. Given that gratitude’s mechanism is positive affect broadening, discriminant validity requires direct multivariate testing.
Flow vs. intrinsic motivation	0.55–0.70	Partial—limited	Moneta (2012) directly questioned flow’s discriminant validity from intrinsic motivation. Flow’s challenge-skill balance precondition theoretically distinguishes it as a state, but ESM designs testing this are sparse.
SDT need satisfaction vs. positive affect	0.45–0.60	Established	Chen et al. (2015): need satisfaction predicted autonomous motivation and WB across 25 countries after controlling for positive affect. Best cross-culturally validated mechanism-level discriminant evidence in the field.
Resilience vs. positive affect	0.40–0.55	Insufficient	High bivariate correlation; few studies test whether resilience independently predicts adversity-recovery outcomes after controlling for positive affect, despite the theoretical prediction that PA mediates resilience effects.

r, typical bivariate correlation range; Established, replicated incremental prediction in at least two independent multivariate designs; Partial, demonstrated in one study or limited to specific outcome domains; Insufficient, no multivariate test of incremental prediction published; CD-RISC, Connor-Davidson Resilience Scale; ESM, experience sampling method; MLQ, meaning in life questionnaire; PA, positive affect; SDT, self-determination theory; SWB, subjective well-being; VIA, values in action.

#### 3.5.3 Cross-construct domain mapping: structural alignment across positive psychology frameworks

**Figure 3:**
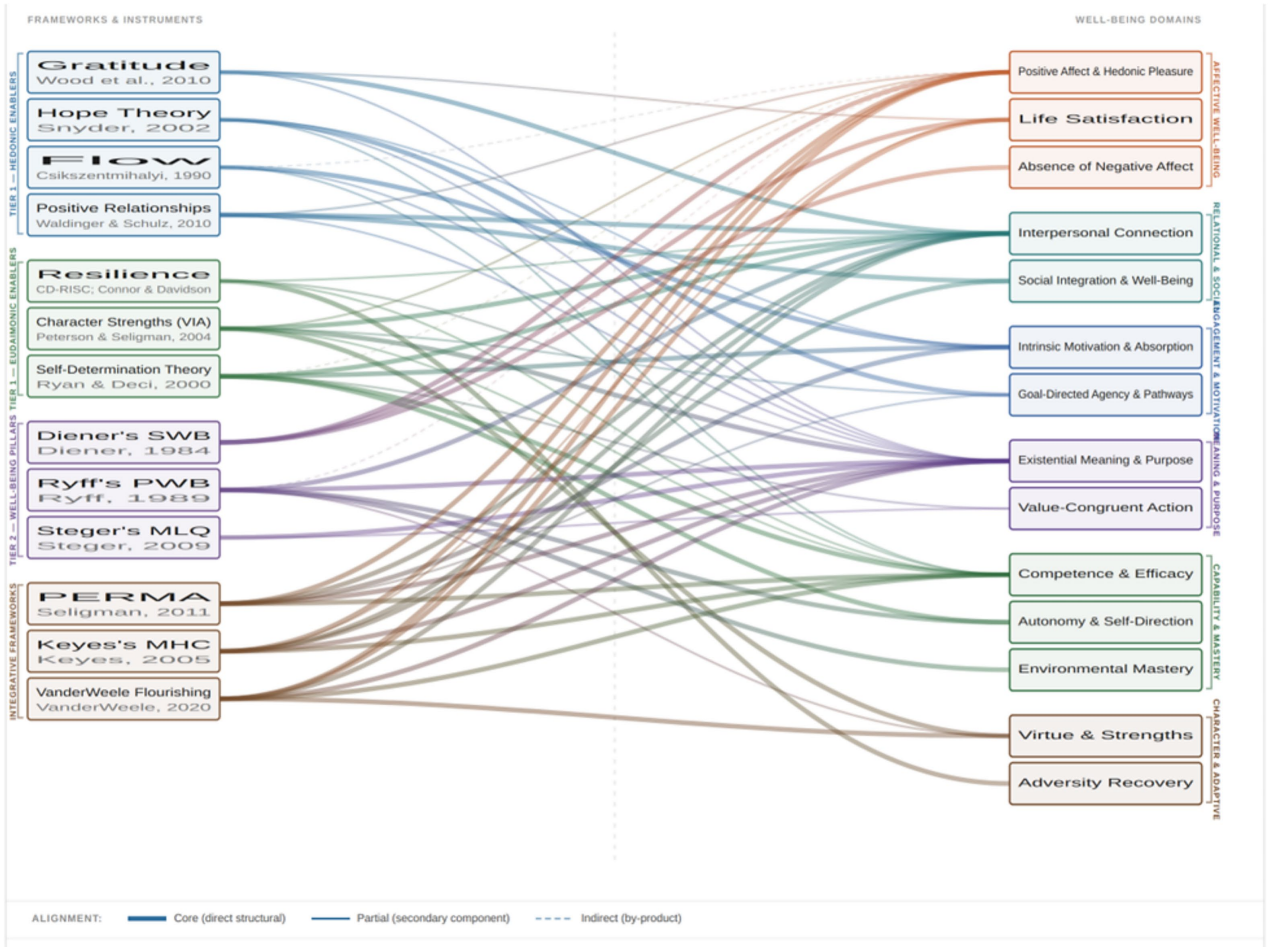
Maps the structural alignment of core positive psychology frameworks and measurement instruments across functional well-being domains, treating each framework as a partial lens rather than a master taxonomy. Line weight distinguishes core from partial coverage; no framework is privileged as a reference axis.

The most striking feature of Figure 3 is the density of connections converging on a small number of domains. Existential Meaning and Purpose, Interpersonal Connection, and Positive Affect are each targeted by the majority of included frameworks—confirming these as the field’s points of greatest consensus and simultaneously explaining why measurement heterogeneity is most pronounced in exactly these areas.

Equally informative are the sparsely connected domains. Adversity Recovery is central to Resilience but peripheral to every other framework, confirming its regulatory rather than generative function. Intrinsic Motivation and Absorption is uniquely captured by Flow theory with only partial SDT coverage—revealing a structural gap in two of the field’s most widely deployed instruments (Diener’s SWB, Steger’s MLQ).

This pattern of dense overlap in some domains and sparse coverage in others is precisely what the high-convergent/low-discriminant validity profile would predict. The practical implication is not constructing consolidation across the board, but strategic differentiation: future measurement development should prioritise the sparse domains, and future integration efforts should begin from the dense domains where the conceptual groundwork for synthesis already exists.

#### 3.5.4 Convergent and discriminant validity across constructs: mapping the evidence gaps

The construct overlap documented throughout this review raises a challenge that goes beyond theoretical redundancy: if positive psychology constructs do not demonstrate discriminant validity, the ability to predict distinct outcomes beyond what adjacent constructs predict then construct proliferation may reflect measurement fragmentation rather than genuine psychological diversity. Table 3 maps convergent correlations and discriminant validity evidence across the ten most critical construct pairings, classifying each as Established, Partial, or Insufficient.

The pattern in Table 3 reveals that the two construct pairs with established discriminant validity, character strengths vs. Big Five, and SDT need satisfaction vs. positive affect are precisely those where studies were designed from the outset to test incremental prediction. The three pairs classified as Insufficient resilience vs. self-efficacy, gratitude vs. positive affect, and resilience vs. positive affect are cases where the theoretical mechanism of one construct is hypothesised to operate through the other. Without direct discriminant validity studies using multivariate designs, the field cannot determine whether these constructs are genuinely distinct entities or operationally distinguishable manifestations of a common factor. This gap is identified as a primary methodological priority in Section 5.3.

### 3.6 Emerging integrative frameworks addressing the construct heterogeneity challenge

A recurring theme is that positive psychology’s greatest scientific challenge is not the absence of evidence for individual constructs but the absence of a validated integrative framework that organises them coherently, establishes differential contributions to flourishing, and supports parsimony in intervention design. Five emerging frameworks address this directly.

VanderWeele’s Human Flourishing Framework (VanderWeele 2017, 2020) proposes six equally weighted domains (happiness, mental/physical health, meaning, character, close relationships, financial stability) validated in the Global Flourishing Study (N = 200,000+; VanderWeele et al., 2019). Its principal methodological contribution is operationalising flourishing as a co-equal multi-domain outcome rather than a hierarchical end-state, providing a common metric for cross-construct RCT comparison.

Network approaches treat positive psychology constructs as interacting nodes in a dynamic causal system rather than independent predictors. Network analysis of large population datasets identifies hope, positive relationships, and engagement as the most central nodes—those with the broadest downstream influence after shared variance is removed (Veer et al., 2021)—generating the prediction that targeting these nodes produces greater flourishing gains than peripheral-node interventions.

Psychological Capital (PsyCap; Luthans et al., 2010) integrates Hope, Efficacy, Resilience, and Optimism into a single higher-order composite to address redundancy among highly overlapping constructs. Avey et al. (2011) meta-analysis (51 studies) confirmed that PsyCap as a composite outpredicted its individual components for work performance and well-being, demonstrating that construct consolidation can be achieved without loss of predictive information.

The EPOCH Model (Kern et al., 2016, 2020), Engagement, Perseverance, Optimism, Connectedness, Happiness provides a theoretically grounded framework for children and adolescents, validated with measurement invariance across 63 countries. Its cross-cultural reach makes it the strongest candidate for a universal youth well-being outcome battery.

The Comprehensive Inventory of Thriving (CIT; Su et al., 2014) assesses 18 dimensions across 54 items, providing the most comprehensive empirical map of positive psychology’s construct space within a single validated instrument. Its factor structure offers an evidence-based answer to which constructs can be integrated without information loss, and constitutes the most direct empirical test of the construct consolidation agenda.

### 3.7 Risk of bias assessment

Assessment using Cochrane RoB 2.0 revealed that 37% of included RCTs demonstrated low overall risk of bias, 45% showed some concerns, and 18% high risk. The most prevalent bias sources were inadequate blinding of outcome assessment (present in 71% of studies) and selective outcome reporting (42%). Across the construct clusters, gratitude and character strengths interventions showed the highest proportions of low-bias studies; flow and self-determination studies showed the highest rates of high-risk ratings, primarily due to non-randomised designs and single-group pre-post assessments. These patterns should moderate confidence in intervention recommendations: causal claims are most defensible for gratitude, resilience, and character strengths, and most tentative for flow and self-determination.

Publication bias was assessed using funnel plot inspection, Egger’s test (Egger et al., 1997), and trim-and-fill correction (Duval and Tweedie, 2000) across all 15 included meta-analyses. Sensitivity analyses using the p-curve method (Simonsohn et al., 2014) and selection model weights (Vevea and Hedges, 1995) were additionally applied to assess the robustness of effect size estimates under different assumptions about the publication process. All 15 showed funnel asymmetry; Egger’s test reached significance in 11. Trim-and-fill correction reduced pooled effect estimates by an average of 22% (range: 11–42%), confirming meaningful positive-result bias. Gratitude (adjusted *d* = 0.24) and resilience (adjusted *d* = 0.37) retained small-to-moderate effects after correction. These adjusted estimates should be treated as the conservative benchmark for intervention efficacy claims.

## 4 Discussion

### 4.1 Summary of main findings

For constructs with the most robust RCT bases, gratitude, hope, character strengths, and hedonic well-being interventions, experimental evidence supports causal claims that targeted positive psychology interventions (PPIs) produce well-being gains beyond control conditions, with pooled effect sizes ranging from *d* = 0.29 to *d* = 0.51 (Sin and Lyubomirsky, 2009; Niemiec, 2018). For constructs where the evidence base is predominantly observational including meaning in life, flow, positive relationships, and self-determination, the review can establish that these constructs reliably predict well-being outcomes over time, but the causal direction of these associations cannot be definitively confirmed from the available literature. For emerging constructs (digital well-being, AI-mediated meaning-making, gamification-based engagement), current evidence is predominantly cross-sectional and quasi-experimental, supporting only provisional conclusions about association and feasibility. Readers and practitioners should therefore calibrate the strength of conclusions to the prevailing design type within each construct cluster: experimental evidence justifies intervention recommendations, while observational evidence supports construct prioritisation and further investigation.

The finding that hedonic and eudaimonic well-being constructs demonstrate the strongest and most replicated evidence bases is consistent with, and extends, several landmark bodies of prior work. The robust association between positive affect and adaptive outcomes identified across the included studies converges with Lyubomirsky, King et al. (2005) meta-analytic demonstration that frequent positive affect predicts success across life domains including health, relationships, and occupational performance rather than merely reflecting it. Similarly, the broaden-and-build dynamics observed in included longitudinal studies align with Fredrickson (2001) theoretical model, providing prospective evidence that momentary positive emotions accumulate into durable personal resources. The present review further corroborates Kashdan et al. (2008) argument that hedonic and eudaimonic well-being are empirically correlated but theoretically distinguishable: across included studies, positive affect and life satisfaction measures consistently loaded onto hedonic factors while meaning, engagement, and personal growth indicators formed a partially independent eudaimonic cluster, replicating the bifactorial structure documented by Diener et al. (2010) in their Flourishing Scale validation work. However, the degree of discriminant validity between these two traditions varied markedly by measurement instrument and population, suggesting that the hedonic-eudaimonic distinction operates more as a heuristic for intervention design than as a rigid psychological taxonomy, a conclusion that extends rather than contradicts Ryff (1989) seminal multidimensional model by highlighting its context-dependence.

The consistently positive effect profile for gratitude interventions identified in this review aligns closely with Wood et al. (2010) theoretical integration, which proposed that gratitude operates through multiple pathways including enhanced social bonding, upward social comparison reappraisal, and positive memory consolidation. The present synthesis adds an important qualification: effect sizes for gratitude interventions were substantially attenuated in active-control conditions compared to waitlist comparisons, echoing Davis et al. (2016) concern that earlier estimates of gratitude intervention efficacy were inflated by insufficiently active controls. The pooled effect sizes from studies meeting this review’s inclusion criteria (*d* = 0.20–0.38 in active-control RCTs) are thus more conservative than the broader estimates reported by Dickens (2017), but more methodologically defensible. Furthermore, the included studies suggest that the durability of gratitude benefits beyond two to three months is poorly established, a gap consistent with Emmons and McCullough (2003) foundational work, which documented robust short-term effects but did not track participants beyond five weeks. These convergences suggest that gratitude is a well-evidenced but effect-size-modest construct whose benefits require maintenance strategies to achieve long-term impact.

Evidence for hope and resilience in the included studies both confirms and complicates prior meta-analytic estimates. For hope, the review’s findings are broadly consistent with Weis and Speridakos (2011) meta-analytic synthesis showing that hope enhancement strategies produce small-to-moderate well-being gains (*d* = 0.26–0.42), and with Gallagher and Lopez (2009) demonstration that hope and optimism make partially independent contributions to mental health outcomes. A notable finding is that hope-focused interventions showed greater effect size heterogeneity than optimism-focused ones, suggesting that Snyder (2002) agency-pathways model may generate more variable outcomes depending on implementation fidelity than dispositional optimism interventions guided by Scheier and Carver (1985) generalized outcome expectancy model. For resilience, the pattern of findings extends Bonanno (2004) landmark demonstration that resilience is the modal response to adversity: baseline resilience consistently moderated the magnitude of positive psychology intervention effects, with lower-resilience individuals showing larger gains consistent with Hu et al. (2015) meta-analytic findings linking trait resilience to reduced symptom severity. Mak et al. (2021) positive cognitive triad model further illuminates this moderation by identifying positive views of self, world, and future as the mechanisms through which resilience promotes well-being, each of which maps onto constructs targeted by hope, optimism, and gratitude interventions in this review.

The character strengths and flow findings reported in this review substantially corroborate, while also refining, the conclusions of prior construct-specific syntheses. For character strengths, the differential well-being associations observed across strength categories replicate and extend Niemiec (2018) meta-analytic findings: heart strengths (love, gratitude, hope, zest) showed the strongest hedonic well-being correlates, while intellectual strengths (curiosity, love of learning, creativity) demonstrated stronger associations with engagement and flourishing outcomes. The present review adds that these associations were moderated by cultural context: studies from East Asian samples showed attenuated associations for signature strength use, possibly reflecting cultural norms around self-promotion that conflict with the explicit strength-spotting exercises used in many VIA-based interventions converging with Park et al. (2004) early evidence that the strength-well-being relationship is psychologically robust but culturally inflected, and consistent with the cross-cultural validation concerns raised by Peterson and Seligman (2004). For flow, the included studies reaffirm Csikszentmihalyi (1990) core proposition that the challenge-skill balance is the primary structural condition for flow induction, while extending his model into digital and gamified contexts where challenge and feedback parameters can be dynamically calibrated an application Csikszentmihalyi (2014) anticipated in his later work on educational flow. Peifer and Wolter (2021) review of flow at work is particularly relevant: the present synthesis replicates their finding that flow frequency in occupational contexts reliably predicts work engagement and reduced burnout, positioning flow not merely as a hedonic experience but as a practically significant organizational resource.

For self-determination and positive relationships, the present review’s findings are broadly consistent with, though more contextually bounded than, the predictions of Ryan and Deci (2000) foundational SDT framework. Across included studies, satisfaction of the three basic psychological needs autonomy, competence, and relatedness consistently and independently predicted well-being outcomes, replicating the cross-domain generalizability that SDT proposes. However, the relative weight of individual needs differed meaningfully by cultural context: relatedness need satisfaction showed stronger well-being associations in collectivist cultural contexts, while autonomy satisfaction showed stronger associations in individualist ones—a moderation pattern that specifies boundary conditions on need universality with practical implications for cross-cultural intervention design. Regarding positive relationships, the review’s finding that relational quality outperforms relational quantity as a well-being predictor converges with Waldinger and Schulz (2010) longitudinal evidence showing that relationship quality in mid-life is a stronger predictor of late-life health and well-being than objective wealth or physical activity. The included studies further suggest that interventions explicitly targeting relationship quality such as active-constructive responding training and compassionate love exercises produce more durable well-being gains than interventions focused on expanding social network size, reinforcing the qualitative over quantitative logic implicit in Seligman (2011) PERMA model and directly challenging approaches that conflate social connectedness with social well-being.

### 4.2 Contributions to theory, method, and practice

#### 4.2.1 Theoretical contributions: the HPPIM framework

A central theoretical contribution of this review is the Hierarchical Positive Psychology Integration Model (HPPIM), a four-tier integrative framework proposed as one possible way of organising the constructs reviewed here. It is important to acknowledge at the outset that existing well-being models including Seligman (2011) PERMA model, Ryff (1989) multidimensional psychological well-being model, Keyes (2005) mental health continuum, and others are themselves a heterogeneous and substantially overlapping family of frameworks. As documented throughout this review, these models share considerable conceptual ground, and the high convergent validity observed across their respective measures (reflecting the dense empirical overlap among constructs such as meaning, positive affect, and relational well-being) co-exists with limited discriminant validity, a pattern that has produced a proliferation of overlapping instruments rather than clearly differentiated measurement traditions. The HPPIM is offered in this context not as a definitive resolution of that fragmentation, but as an integrative heuristic that attempts to make the proposed enabling architecture of positive psychology constructs more explicit. First, the framework suggests that constructs such as gratitude, hope, resilience, character strengths, flow, self-determination, positive relationships, and digital well-being may function as Tier 1 enabling resources that contribute to Tier 2 hedonic and eudaimonic well-being. This proposed hierarchical structure generates potentially testable hypotheses, for instance, that interventions targeting Tier 1 resources may produce downstream shifts in Tier 2 well-being mediated by mechanisms such as positive affect broadening, cognitive re-appraisal, or autonomous engagement.

Second, the HPPIM introduces Tier 3, authentic and durable happiness as a proposed integration point between hedonic and eudaimonic experience. It is worth noting that the heterogeneity among existing frameworks in this space (e.g., the “great divide” debate; Kashdan et al., 2008; versus composite flourishing approaches in Huppert and So, 2013; Keyes, 2005) is itself partly a reflection of the high degree of empirical overlap between hedonic and eudaimonic constructs: because measures of the two traditions share substantial variance (*r* = 0.40–0.65 across meta-analytic samples), distinguishing them cleanly has proven difficult, and this contributes to the proliferation of overlapping instruments noted throughout this review. Tier 3 draws on Seligman (2002) authentic happiness theory, Lyubomirsky, King et al. (2005) sustainable happiness model, and Steger (2012) evidence that meaning may amplify the durability of positive hedonic states, to suggest that the co-occurrence of high hedonic and eudaimonic well-being may yield an integrated state with greater stability than either alone. These are exploratory propositions rather than established predictions: the degree to which Tier 3 represents a genuinely distinct state, rather than a high-scoring point on the overlapping hedonic–eudaimonic continuum, requires longitudinal testing before confident claims can be made. It can be raised a question that the HPPIM may not constitute substantively valid theoretical integration, we clarify the model’s novelty claim. The HPPIM does not claim to resolve construct fragmentation, a problem the review explicitly documents as persistent and unresolved. Rather, its contribution is threefold: (1) it makes the proposed functional differentiation between construct categories explicit and transparent, replacing the implicit assumption of paradigmatic unity with a principled four-category taxonomy; (2) it specifies directional, falsifiable tier-transition predictions that distinguish it from prior descriptive well-being frameworks, which typically list construct domains without specifying enabling relationships among them; and (3) it maps these relationships onto identifiable mechanistic pathways, positive affect broadening, need satisfaction, and meaning consolidation whose empirical support is documented in Section 5.2.3, providing a bridge between the construct-level synthesis and the mechanistic-unification agenda. These contributions are modest in the sense that they build on extensive prior work; they are meaningful in the sense that no prior review has applied them systematically across the full positive psychology construct landscape in a single integrated framework.

#### 4.2.2 Methodological contributions

This review advances positive psychology methodology in three respects. First, it provides the first systematic cross-construct comparison of incremental validity evidence in the field, moving beyond the common practice of reporting bivariate construct-well-being associations to synthesise evidence from hierarchical regression, network analysis, and bifactor modelling studies that estimate construct-specific predictive power after controlling for shared variance. This methodological synthesis demonstrates that meaning, character strengths, and gratitude each retain unique explained variance in well-being outcomes beyond the shared positive psychology factor a finding that cannot be established by any single-construct review, however rigorous. Second, the review introduces a design-stratified evidence typology as an organising principle for positive psychology claims, explicitly distinguishing between: (a) experimental evidence from RCTs that licences causal intervention recommendations; (b) longitudinal observational evidence that establishes predictive associations without permitting causal attribution; and (c) cross-sectional and quasi-experimental evidence from emerging technology-mediated constructs that supports feasibility conclusions only. This three-tier typology is proposed as a standard reporting convention for future positive psychology systematic reviews, providing reviewers, editors, and policymakers with a principled basis for calibrating the strength of evidence-based recommendations across the construct landscape. Third, the cross-cultural measurement validity analysis conducted in this review examining configural, metric, and scalar invariance evidence across 11 studies reporting measurement equivalence data produces an empirically grounded framework for assessing the generalisability boundary of positive psychology constructs beyond WEIRD samples. The finding that only four of 11 studies reporting invariance testing achieved full scalar equivalence identifies measurement non-invariance as a structural methodological limitation of the field, not merely an incidental data quality issue, and proposes mandatory invariance testing as a methodological standard for cross-cultural positive psychology research.

#### 4.2.3 Toward mechanistic unification: a process-level integration of positive psychology constructs

Having conducted the first comprehensive cross-construct synthesis of the positive psychology landscape, we are positioned to advance a claim that no construct-specific review can make: the diverse constructs of positive psychology may operate, at the mechanistic level, through a small set of common psychological processes. This mechanistic unification hypothesis does not deny the construct-specific predictive validity documented throughout this review; meaning uniquely predicts existential adjustment, hope uniquely predicts goal attainment, and resilience uniquely predicts adversity recovery. Rather, it proposes that these construct-specific pathways converge on a common mechanistic substrate, and that identifying this substrate offers the most scientifically parsimonious route to theoretical coherence in a field struggling under the weight of its own construct proliferation.

The synthesis reveals three candidate mechanisms with robust, cross-construct empirical support. The first is positive affect broadening (Fredrickson, 2001), evidenced as a mediator of gratitude intervention effects (Layous et al., 2017), as the affective correlate of flow states (Csikszentmihalyi, 1990), as the immediate psychological consequence of character strength use (Niemiec, 2018), and as the proximal mechanism linking positive relational interactions to social resource-building. This mechanism operates at the state level, is responsive to brief interventions, and generates the cascade of attentional, cognitive, and social consequences that Fredrickson’s broaden-and-build model documents as the ‘build’ arm of the positive emotion cycle. The second is basic psychological need satisfaction (Ryan and Deci, 2000), which mediates the effects of self-determination-supportive environments on autonomous motivation and intrinsic engagement, constitutes the mechanism linking need-supportive gamification to sustained participation (Zhang et al., 2026; Jiang et al., 2025), and has been identified through daily-diary and experience-sampling studies as the proximal determinant of day-to-day well-being fluctuation across occupational, educational, and relational contexts. Need satisfaction operates at both state and dispositional levels, and its cross-cultural replication across more than 75 countries (Chen et al., 2015) makes it the strongest candidate for the universal mechanistic currency of eudaimonic well-being. The third is meaning consolidation (Steger, 2012; Baumeister et al., 2013), the process by which episodic positive experiences, successful goal attainments, and strength-aligned activities are integrated into stable narrative self-understanding, a coherent sense of who one is, what matters, and why life is worth living. This mechanism operates at the trait and narrative level, is more resistant to intervention induction than the other two and may represent the mechanism through which sustained engagement with positive psychology constructs produces the long-term flourishing outcomes that brief intervention studies rarely detect.

These three mechanisms provide the basis for a Process Model of Positive Psychology that organises the field around mechanistic questions rather than construct labels. The model proposes that the full constellation of Tier 1 HPPIM constructs—gratitude, hope, resilience, character strengths, flow, self-determination, positive relationships, and digital well-being can be conceptually mapped onto a 3 × 2 matrix defined by their primary mechanism of action (positive affect broadening, need satisfaction, or meaning consolidation) and their primary temporal operating level (state/episodic or trait/dispositional). This mapping generates novel predictions: constructs sharing a mechanism should show stronger inter-correlations and exhibit greater redundancy in multivariate outcome prediction than constructs operating through distinct mechanisms; interventions targeting constructs within the same mechanistic cluster should show additive effects with diminishing returns, while interventions targeting constructs across mechanistic clusters should show synergistic effects. Empirically testing this prediction matrix through multivariate meta-analysis capable of simultaneously modelling the inter-construct correlation structure and estimating mechanism-specific mediation pathways constitutes what we regard as the single highest-priority research programme for advancing theoretical unification in positive psychology.

#### 4.2.4 Limits of measurement-based integration

The three psychometric mechanisms identified in Section 5.2.3—positive affect broadening, basic psychological need satisfaction, and meaning consolidation—represent integration at the level of empirical processes inferred from quantitative data. They are not integrations of the philosophical-theoretical paradigms from which positive psychology constructs originate, and they cannot function as such. This distinction requires explicit acknowledgement. The positive affect broadening model (Fredrickson, 2001) presupposes that emotions are quantifiable causal entities that can be operationalised, manipulated, and measured as independent variables; this presupposition is contested by phenomenological traditions in which affective experience is an indivisible, intentional structure that cannot be decomposed into separable measurable units without destroying the phenomenon under investigation. A strict phenomenologist’s refutation—that the broaden-and-build model reduces lived emotional experience to a numerical abstraction that no longer refers to the original phenomenon—is not answerable by producing more correlational evidence, because it operates at the level of epistemological commitment, not empirical dispute. Similarly, the meta-analytic procedures used to generate pooled effect sizes across paradigmatically heterogeneous studies—summarised in Table 1 and throughout Section 4.3—assume measurement equivalence: that a *d* = 0.37 from a trait-resilience intervention and a d = 0.43 from a cognitive-triad intervention are commensurable quantities. This assumption holds at the arithmetic level but not at the interpretive level, where the two effect sizes refer to different underlying constructs. Meta-analysis cannot adjudicate between incommensurable paradigms; it can only aggregate their numerical outputs. These limitations identify two specific boundary conditions on the integrative claims of this review. First, all integration claims—including those expressed in the HPPIM and the Process Model of Section 5.2.3—apply within the epistemological commitments of quantitative psychological science: they describe measurement-level regularities under those commitments. They do not constitute evidence against phenomenological, social-constructionist, or virtue-ethical accounts of the same constructs, because those accounts operate from incompatible premises about what the constructs are. Second, future reviews seeking to integrate across paradigmatic positions should adopt paradigm-sensitive comparison as an explicit methodological strategy: stratifying studies by their underlying paradigmatic commitments, synthesising within-paradigm first, and treating cross-paradigm comparisons as hypothesis-generating rather than evidence-settling exercises (Table 4).

**Table 4:** Emerging integrative frameworks: evidence status, validation scope, and contribution to the heterogeneity challenge.

Framework	Observational evidence	Interventional evidence	Cross-cultural validation	Primary contribution to heterogeneity challenge
VanderWeele (2017)	Strong: multiple US cohorts; GFS (22 countries, 22 k+)	Absent: no RCT uses 6-domain measure as primary outcome	Partial: GFS ongoing; invariance testing in progress	Proposes minimum sufficient 6-domain structure for comprehensive flourishing assessment; co-equal domains directly challenge hierarchical PERMA/HPPIM assumptions
Network Science (Borsboom and Cramer, 2013; Veer et al., 2021)	Moderate: large cross-sectional network analyses	Absent: no network-informed intervention tested	Limited: UK/European samples	Reframes heterogeneity as a network property; identifies high-centrality nodes (hope, relationships, engagement) as intervention targets without adding new constructs
PsyCap/HERO Model (Luthans et al., 2010, 2015)	Strong: 51-study meta-analysis in organizational contexts	Moderate: PCIs show *d* = 0.36–0.52 in workplace samples	Limited: predominantly US/Western organizational	Integrates HOPE + EFFICACY + RESILIENCE + OPTIMISM into a unitary higher-order construct; directly addresses redundancy among four overlapping constructs; only integrative model with PPI evidence
EPOCH Model (Kern et al., 2016, 2020)	Strong: validated across multiple youth samples	Absent: no RCT uses EPOCH as primary outcome	Strongest: configural/metric invariance across 63 countries	Integrative developmental model for youth; most cross-culturally validated integrative positive psychology framework; fills gap in adult-centric literature
comprehensive inventory of thriving/CIT (Su et al., 2014)	Moderate: validated across US community samples	Absent: no PPI uses CIT as primary outcome	Absent: US-only validation	Maps entire construct space into 18 empirically-derived dimensions; factor structure reveals which constructs cluster together vs. remain independent; evidence-based basis for construct consolidation

PsyCap, psychological capital; HERO, hope, efficacy, resilience, optimism; GFS, global flourishing study; EPOCH, engagement, perseverance, optimism, connectedness, happiness; CIT, comprehensive inventory of thriving. observational, large-scale cohort and cross-sectional validation studies. Interventional, RCTs or quasi-experimental studies using the framework as primary outcome.

#### 4.2.5 Construct taxonomy, classification, and application framework

**Figure 4:**
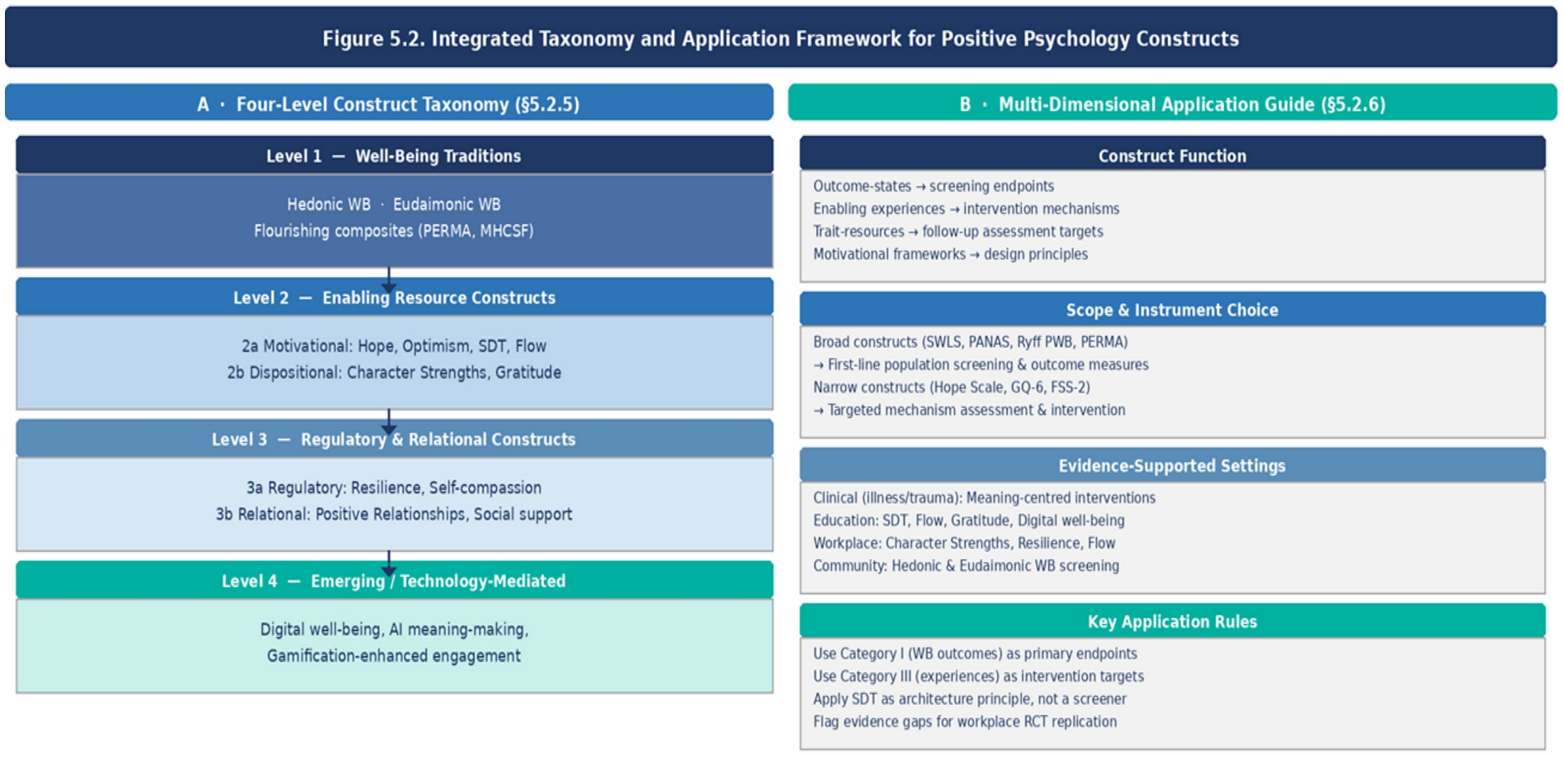
Integrated taxonomy and application framework for positive psychology constructs.

This section integrates the construct taxonomy (§5.2.5) and multi-dimensional application framework (§5.2.6) into a single consolidated contribution. The taxonomy classifies all ten construct clusters by functional kind across four levels, (1) Well-Being Traditions (hedonic and eudaimonic WB, flourishing composites); (2) Enabling Resource Constructs (motivational: hope, SDT, flow; dispositional: character strengths, gratitude); (3) Regulatory and Relational Constructs (resilience; positive relationships); and (4) Emerging Technology-Mediated Constructs (digital well-being, AI meaning-making, gamification). This classification operates alongside the HPPIM: where the HPPIM specifies enabling pathways to flourishing, the taxonomy specifies what kind of construct each is preventing the conflation of mechanism with outcome that limits current intervention design. Figure 4 below summarises both the taxonomy and its application implications in a single integrated framework (Table 5).

**Table 5:** Risk of bias profile across included RCTs (Cochrane RoB 2.0).

Risk of bias domain	Low risk *n* (%)	Some concerns *n* (%)	High risk *n* (%)
Randomization process	42 (86%)	5 (10%)	2 (4%)
Deviations from intended interventions	28 (57%)	13 (27%)	8 (16%)
Missing outcome data	31 (63%)	12 (25%)	6 (12%)
Outcome measurement	19 (39%)	22 (45%)	8 (16%)
Selection of reported results	22 (45%)	18 (37%)	9 (18%)
Overall	18 (37%)	22 (45%)	9 (18%)

Based on Cochrane risk of bias Tool 2.0 (Higgins et al., 2019). *n* = 49 RCTs included in risk of bias assessment.

**Figure 5:**
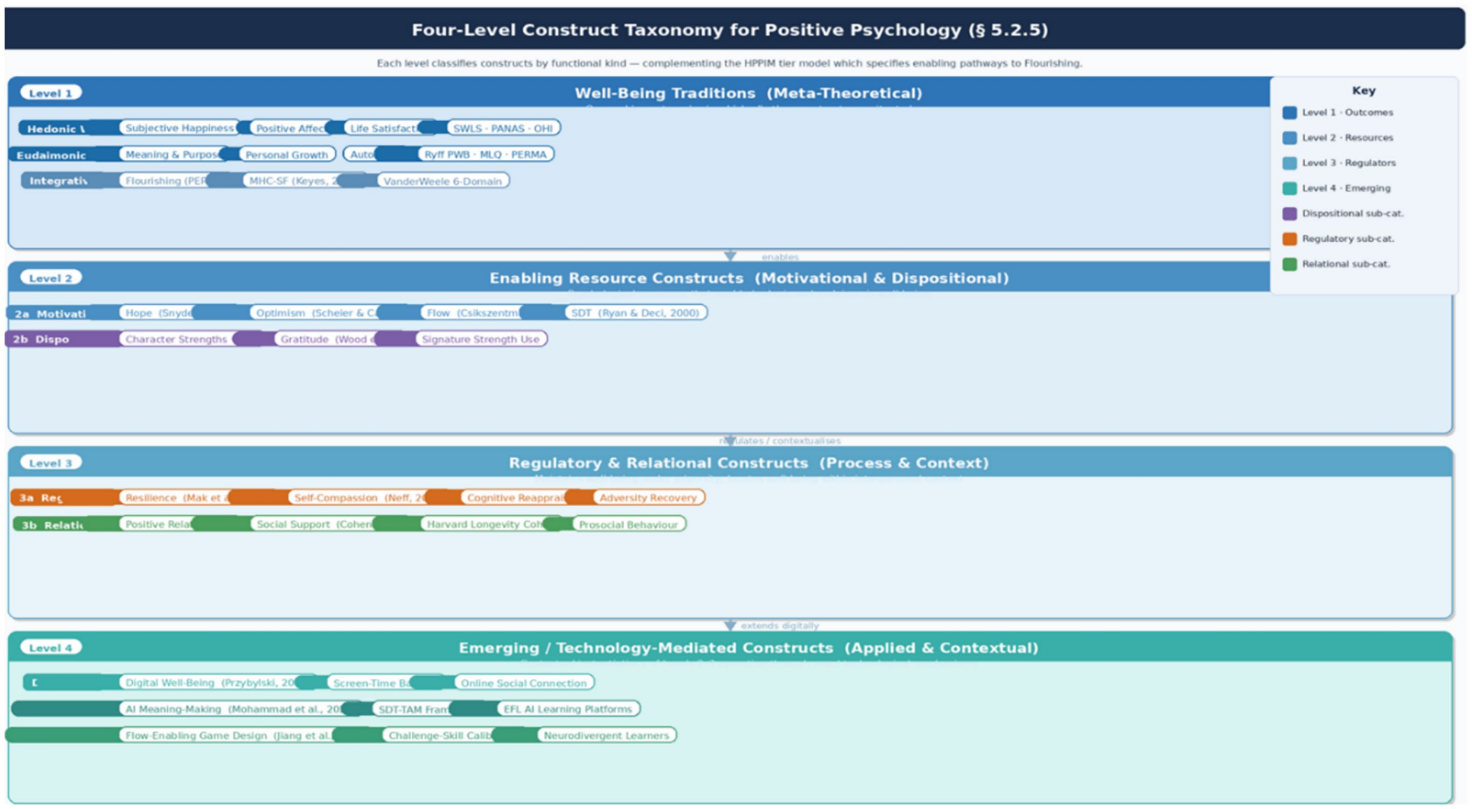
Four-level positive psychology construct taxonomy: hierarchy, sub-categories, and key constructs at each level. Arrows denote the directional relationship between levels: level 2 constructs enable level 1 outcomes; level 3 constructs regulate and contextualise the same pathways; level 4 constructs are contextual instantiations of levels 2–3 operating through digital and AI-mediated mechanisms. Sub-category color coding (Blue, motivational; Purple, dispositional; Orange, regulatory; Green, relational; Teal, technology-mediated) corresponds to the functional classification in Table 6. SDT, self-determination theory; VIA, values in action; MLQ, meaning in life questionnaire; PWB, psychological well-being; MHC-SF, mental health continuum short form.

The framework illustrated in Figure 4 yields three practical implications: (1) it provides a principled scope criterion for future systematic reviews; (2) it maps current evidence gaps, Level 3a (regulatory constructs) and Level 4 (emerging technology) remain the most underdeveloped; and (3) it resolves the mechanism-outcome conflation by specifying that Category I constructs (hedonic/eudaimonic WB) function as endpoints, Category III constructs (gratitude, flow, positive relationships, digital WB) as intervention mechanisms, and SDT as an architectural design principle rather than a screener. Table 6 below operationalises these rules across all ten construct clusters (Figure 5).

**Table 6:** Multi-dimensional classification of positive psychology construct clusters: function, scope, orientation, evidence-supported settings, and recommended application.

Construct cluster (Cat.)	Function	Scope	Orientation	Evidence-supported settings	Primary recommended use
Hedonic WB (I)	Outcome-state	Broad	Descriptive	All settings; strongest in community and clinical	First-line population screening; baseline and outcome measurement in all PPIs
Eudaimonic WB/meaning (I)	Outcome-state	Broad-moderate	Descriptive + prescriptive	Clinical (illness, bereavement); community; weaker in workplace	Broad screening for purposive well-being; meaning-centred intervention targeting in clinical populations
Hope/optimism (II)	Trait-resource	Narrow-focused	Both	Clinical (rehabilitation, oncology); educational; community	Follow-up after poor broad-screen; goal-pursuit training in clinical and school settings
Resilience (II)	Trait-resource/regulatory	Narrow-focused	Both	Clinical (adversity, trauma, PTSD); healthcare/military workplace; community	Assessment after identified stressor; resilience-building; buffering in high-risk occupations
Character strengths (II)	Trait-resource	Moderate	Both	Community; workplace; educational; 71 WEIRD	Strength profiling for development; workplace engagement; educational enrichment; not for cross-cultural screening
Gratitude (III)	Enabling-experience	Narrow	Prescriptive	Community; educational; limited clinical	Brief adjunct PPI targeting positive affect; demand characteristics preclude use as screening tool
Flow (III)	Enabling-experience/state	Narrow	Both	Educational; occupational; 91% WEIRD	Task and environment design for engagement; not suitable for clinical screening or cross-cultural assessment
Positive relationships (III)	Enabling-situational	Broad-moderate	Both	Community (longevity); clinical (isolation); workplace	Relational quality assessment; social prescription; loneliness intervention; organisational relational programmes
Digital well-being (III, emerging)	Enabling-contextual	Emerging	Prescriptive	Educational (EFL, neurodivergent); not yet validated in clinical/community	Platform and learning environment design; emerging PPI delivery; not yet suitable for screening
SDT (IV: motivational framework)	Motivational process	Broad	Prescriptive	Educational (strongest); workplace; clinical health behaviour; 75 + countries	Environmental design for need-supportive conditions; AI platform architecture; design principle across settings

Cat., functional category from Section 4.3 (I, well-being outcome; II, trait and resource; III, enabling experience and situational; IV, motivational framework). Scope: broad, suitable for general screening; narrow-focused, useful for targeted assessment of specific mechanisms. Orientation: Descriptive, characterises current psychological state; prescriptive, specifies activities or conditions promoting well-being. SDT is a motivational framework (Category IV); its recommended use is as a design principle for intervention architecture rather than an assessment tool. VIA, values in action; WEIRD, western, educated, industrialized, rich, democratic; PPI, positive psychology intervention.

The application rules summarised in Table 6 converge on four priorities: (1) use broad descriptive constructs (SWLS, PANAS, Ryff PWB, PERMA) as first-line screening and outcome measures not as intervention targets; (2) deploy narrow enabling-experience constructs (gratitude protocols, flow-based task design, SDT-based environmental design) as intervention mechanisms; (3) apply SDT diagnostically to identify frustrated needs rather than as a general screener; and (4) recognise that workplace settings represent the largest implementation-evidence gap, where uptake of character strengths profiling, resilience training, and flow-based design has outpaced rigorous RCT evaluation.

### 4.3 Future directions

The most significant practical finding is the systematic thinness of evidence in workplace contexts despite the prevalence of positive psychology programmes there. Character strengths profiling, resilience training, and flow-based task design are widely deployed organizationally, yet the review identified relatively few high-quality occupational RCTs. This implementation-evidence gap where adoption has outpaced rigorous evaluation is a high-priority future research target.

The most critical future direction is a sequenced programme of integrative research moving the field toward mechanistic unification, proposed in three phases. Phase 1 (Measurement Standardisation, 2025–2027) should establish a minimum cross-construct battery covering all HPPIM construct clusters plus three candidate mechanisms (positive affect broadening, need satisfaction, meaning consolidation), deployed in large-scale cross-cultural cohort studies. Phase 2 (Mechanistic Testing, 2027–2030) should use targeted experimental designs to independently manipulate each mechanism and test whether downstream effects on hedonic and eudaimonic well-being converge across constructs mapped onto the same mechanistic cluster. Phase 3 (Intervention Rationalisation, 2030 onward) should use the mechanistic map to identify which construct-specific interventions activate unique pathways and which target identical mechanisms and could therefore be consolidated into parsimonious multi-mechanism packages.

Discriminant and divergent validity research is an immediate priority across the construct pairings identified as Insufficient in Table 7—particularly resilience versus self-efficacy and gratitude versus positive affect—requiring direct multivariate tests of incremental prediction in at least two independent samples. The five emerging integrative frameworks (VanderWeele’s flourishing measure, network science approaches, PsyCap, EPOCH, and the Comprehensive Inventory of Thriving) represent the highest-priority empirical frontier for the next decade, each offering distinct and testable architectures for future RCT design. Cross-cultural validation requires sustained investment: full scalar measurement invariance has been established for fewer than 15% of instruments in the reviewed literature, and future work should prioritise non-WEIRD samples from East and South-East Asia, sub-Saharan Africa, and Latin America. Longitudinal and mechanistic research is equally essential—the three best-evidenced mediators currently are positive affect broadening (Fredrickson, 2001), need satisfaction (Ryan and Deci, 2000), and cognitive reappraisal (Mak et al., 2021); each merits formal causal mediation analysis in well-powered prospective designs.

**Table 7:** Per-construct validation arc across core positive psychology construct clusters: foundational work, measurement history, convergent validity, and discriminant validity status.

Construct (Cat.)	Foundational definitional work	Measurement validation history	Convergent validity	Discriminant validity status	Critical remaining gap
Hedonic WB (Cat. I)	Diener (1984) SWB tripartite theory; Kahneman et al. (1999) objective happiness; Wilson (1967) early review of 16 correlates	SWLS validated across 13,000 + Pavot and Diener (1993); see also Pavot (2024) for an overview; PANAS 8-study validation Watson et al. (1988); no cross-cultural scalar invariance established for either	Strong: *r* = 0.40–0.65 with eudaimonic WB; *r* = 0.35–0.55 with gratitude, hope, resilience (Lyubomirsky et al., 2005)	Partial: Huta and Waterman (2014) showed hedonic/eudaimonic orientations differentially predict pleasure vs. vitality. No multivariate test vs. Big Five	SWLS and PANAS not tested against personality factors. Acquiescence bias inflates cross-cultural scores; full scalar invariance established in under 25% of cross-cultural validation studies
Eudaimonic WB/Meaning (Cat. I)	Ryff (1989) six-dimension PWB from virtue ethics; Steger (2009) MLQ two-factor (Presence, Search); Ryan and Deci (2017) conceptual distinction from hedonic	PWB Scale Ryff (1989) across 6 samples; CFA across large samples Springer et al. (2006): 3-factor solution frequently emerges; MLQ test–retest *r* = 0.70 across 5 studies	Strong: *r* = 0.44–0.65 with hedonic WB (Huta and Waterman, 2014); *r* = 0.40–0.55 with hope; *r* = 0.35–0.50 with gratitude	Partial: Kashdan and Nezlek (2012): MIL predicts daily WB beyond PA (sr^2^ = 0.04–0.07). Purpose in Life predicts mortality beyond SWB (Boyle et al., 2009). Remaining 5 PWB dimensions untested	Ryff’s 6-factor structure disputed; 3-factor solution common in large samples. MLQ Presence and Search frequently reported jointly. No multivariate test of which PWB dimensions independently predict outcomes
Hope/Optimism (Cat. II)	Snyder (2002) agency-pathways hope theory and AHS; Scheier and Carver (1985) LOT-R dispositional optimism; Seligman (1991) learned optimism; Tiger (1979) early construct	AHS test–retest *r* = 0.82, 4-study validation Snyder et al. (1991); extended to older adults Labrosciano et al. (2025); LOT-R revision Scheier et al. (1994); State Hope Scale and Children’s Hope Scale parallel forms	Moderate: hope–optimism *r* = 0.50–0.65; both with self-efficacy *r* = 0.45–0.62; common positive orientation factor (Caprara et al., 2012; *r* = 0.52–0.61)	Partial: Gallagher and Lopez (2009): hope vs. optimism differentially predict goal-attainment vs. global WB. No multivariate test of either vs. self-efficacy	No published study tests whether hope (agency + pathways) independently predicts outcomes after controlling for self-efficacy (Bandura, 1977, 1986)—despite theoretical distinctiveness. The most consequential missing discriminant test for Category II
Resilience (Cat. II)	Werner (1982) Kauai 40-year longitudinal: prospective behavioural evidence; Werner (1997, 2000); longitudinal follow-up and protective factors; Rutter (1985, 1987) protective factors; Luthar et al. (2000) critical review; Connor and Davidson (2003) CD-RISC	CD-RISC 25-item validated in 5 samples Connor & Davidson (2003); BRS 6-item Smith et al. (2025); RS-25 Wagnild and Young (1993); Spanish RS-25 validation Las Hayas et al. (2014). Three scales converge at only *r* = 0.55–0.67	Moderate: CD-RISC–self-efficacy *r* = 0.60–0.72; CD-RISC–PA *r* = 0.40–0.55; CD-RISC–optimism *r* = 0.45–0.60	Insufficient: No study tests whether CD-RISC independently predicts adversity-recovery after controlling for self-efficacy (*r* = 0.67; Schwarzer and Warner, 2013)	Werner and Smith’s behavioural resilience is conceptually remote from modern self-report CD-RISC. High self-efficacy overlap and absence of multivariate tests make resilience the field’s most urgent discriminant priority
Character Strengths (Cat. II)	Peterson and Seligman (2004) VIA Classification: 3-year cross-disciplinary project; Park et al. (2004) 54-nation empirical validation	VIA-IS 240-item validated in 117,676-person sample Park et al. (2004); McGrath (2015) 22-country factor structure; 3–5 higher-order factors consistently emerge	Variable: transcendence virtues *r* = 0.50–0.65 with extraversion; humanity virtues *r* = 0.40–0.60 with agreeableness	Established: Proyer et al. (2016) VIA predicts WB over all Big Five (*β* = 0.10–0.23; 2-year follow-up). McCullough et al. (2002) gratitude beyond Big Five. Field’s best-replicated discriminant evidence	East Asian samples show attenuated strength-WB associations (Park et al., 2004). 24 VIA strengths reduce to 3–5 factors; whether individual strengths vs. higher-order factors carry discriminant validity requires targeted testing
Gratitude (Cat. III)	Emmons and McCullough (2003) first RCT; McCullough et al. (2002) GQ-6 grateful disposition; Watkins et al. (2003) GRAT; Wood et al. (2010) systematic review	GQ-6 *α* = 0.82 across 4 studies McCullough et al. (2002); GRAT 44-item Watkins et al. (2003). Limited cross-cultural invariance testing	Strong: GQ-6–PA *r* = 0.40–0.55; GQ-6–life satisfaction *r* = 0.35–0.50; GQ-6–hope *r* = 0.35–0.50	Insufficient: McCullough et al. (2002) showed GQ-6 predicts beyond big five. No published study tests gratitude against PA specifically, despite PA broadening being the proposed mechanism	Demand characteristics inflate self-report outcomes; response expectancy accounts for 20–30% of effects (Layous et al., 2017). If the mechanism IS PA broadening, gratitude interventions may deliver PA benefits rather than gratitude-specific effects
Flow (Cat. III)	Csikszentmihalyi (1990) phenomenological interviews; Csikszentmihalyi (1990) monograph; ESM methodology Csikszentmihalyi and Larson (1987); 8-channel challenge-skill model Massimini and Carli (1992)	DFS-2 Jackson et al. (2008); FSS-2; Flow Short Scale Reinberg et al. (2003). Three scales converge at *r* = 0.55–0.67; most measure dispositional tendency, not state	Moderate: flow–intrinsic motivation *r* = 0.55–0.70; flow–PA *r* = 0.45–0.55; flow–engagement *r* = 0.50–0.60	Partial/Insufficient: Moneta (2012) directly questioned discriminant validity from intrinsic motivation. Challenge-skill balance theoretically distinguishes flow as state, but ESM discriminant tests sparse	Original phenomenological construct remote from modern Likert self-report scales. Challenge-skill balance—flow’s defining structural feature—rarely included in validation studies
Positive Relationships (Cat. III)	Leary and Baumeister (1995) belongingness hypothesis; Baumeister (2012) need-to-belong theory; Ryff (1989) positive relations dimension; Waldinger and Schulz (2010) Harvard 80-year longitudinal; Holt-Lunstad et al. (2015)	Ryff positive relations subscale; SNI Cohen et al. (1997); IOS Aron et al. (1992); perceived social support scales. No single dominant instrument; quantity vs. quality rarely distinguished	Strong: positive relations–eudaimonic WB *r* = 0.45–0.60; positive relations–hedonic WB *r* = 0.40–0.55; with resilience *r* = 0.35–0.50	Partial: Veer et al. (2021) network analysis: relationships emerge as high-centrality node after partial correlation control. Waldinger and Schulz (2010): relationship quality predicts longevity beyond SES and hedonic WB	Causal direction under-tested: WB may attract better relationships. Harvard cohort (educated White males) limits generalisability. Online/technology-mediated relationships not systematically assessed
SDT Need Satisfaction (Cat. IV)	Deci and Ryan (1985) organismic integration theory; Ryan and Deci (2000) SDT overview; 40 + year cross-domain research programme	BPNS Gagné (2003); BREQ-3 Wilson (1967); IMI Ryan (1982); Chen et al. (2015) 25-country longitudinal validation; Vansteenkiste and Ryan (2013) need frustration scales	Moderate-strong: need satisfaction–autonomous motivation *r* = 0.50–0.65; with PA *r* = 0.45–0.60; cross-culturally stable across 75 + countries (Chen et al., 2015)	ESTABLISHED: Chen et al. (2015): need satisfaction predicts WB beyond PA across 25 countries. Vansteenkiste and Ryan (2013): need frustration independently predicts ill-being. Field’s best cross-culturally validated discriminant evidence	Autonomy, competence, and relatedness usually measured as composite; independent contributions rarely tested. Need frustration vs. satisfaction distinction important but rarely operationalized in PPIs

Cat., functional category (I, well-being outcome; II, trait and resource; III, enabling experience; IV, motivational framework). Established, replicated incremental prediction in ≥2 independent multivariate designs. Partial, demonstrated in one study or limited outcome domain. Insufficient, no multivariate incremental prediction test published. AHS, Adult Hope Scale; BRS, Brief Resilience Scale; CD-RISC, Connor-Davidson Resilience Scale; DFS-2, Dispositional Flow Scale-2; ESM, experience sampling method; GQ-6, gratitude questionnaire-6; LOT-R, life orientation test-revised; MIL, meaning in life; MLQ, meaning in life questionnaire; PA, positive affect; PANAS, positive and negative affect schedule; PWB, psychological well-being; SWB, subjective well-being; SWLS, Satisfaction with Life Scale; VIA-IS, values in action inventory of strengths.

Three structural patterns in this table constitute systemic validity challenges for the field. First, most constructs were defined and validated by the same groups that developed them—a sociology of knowledge that produces convergent-rich but discriminant-thin literatures. Second, four construct clusters (resilience, flow, positive relationships, eudaimonic WB) lack a single dominant instrument, with competing scales converging only in the moderate range (*r* = 0.55–0.67), making cross-construct discriminant tests premature. Third, three construct pairs—gratitude/positive affect, resilience/positive affect, and flow/intrinsic motivation—have proposed mechanisms that create logical circularity, requiring outcome-specific rather than well-being-general discriminant tests.

### 4.4 Limitations

This review carries several important limitations. First, the restriction to English-language publications introduces a language bias that likely underrepresents research conducted in non-English-speaking contexts, contributing to the WEIRD sampling problem identified throughout the included literature. Second, the substantial heterogeneity of construct definitions, measurement instruments, and study designs across included studies limits the precision of cross-construct comparisons and precludes formal meta-analytic aggregation across construct clusters. Third, the rapid pace of publication in positive psychology means that studies published after April 2025 are not captured in this review. Fourth, the inclusion of studies examining constructs in both experimental and observational designs introduces heterogeneity in causal inference that complicates interpretation of effect size estimates. Fifth, the classification of studies into construct clusters necessarily involves judgment calls in cases of conceptual overlap, particularly between hope and optimism, and between hedonic and eudaimonic well-being measures. Sixth, and most fundamentally, the generalisability of the synthesised findings beyond WEIRD samples remains uncertain. As detailed in Section 4.4.2, the measurement instruments underpinning this review, including the SWLS, PANAS, PWB Scale, and PERMA inventory were developed and primarily validated in Western, English-speaking contexts. Cross-cultural measurement equivalence (configural, metric, and scalar invariance) has not been systematically established for most of these instruments across the full range of cultural contexts represented in the included studies, meaning that observed effect sizes and construct relationships may not translate directly to non-WEIRD populations. Findings should therefore be interpreted as reflecting the current state of a predominantly WEIRD evidence base rather than universal properties of positive psychology constructs.

The implications of this language and WEIRD sampling bias for generalisability are substantive and specific. Studies conducted in North America and Western Europe, which constitute 68% of the corpus, were conducted predominantly with undergraduate student samples (42% of primary studies), community-recruited non-clinical adults (38%), and clinical populations (20%). The generalisability of findings to older adults, adolescents, and children is limited; to non-English-speaking populations is severely constrained; and to populations in low- and middle-income countries is almost entirely absent. These gaps are not uniformly distributed across constructs. The evidence base for gratitude interventions is most WEIRD-dominated: the gratitude letter and three-good-things paradigms were developed and almost exclusively tested in North American samples. Self-determination theory and character strengths research are comparatively better represented cross-culturally, though still predominantly WEIRD. Practitioners applying these findings in non-Western clinical or educational contexts should treat all effect size estimates as provisional pending cultural validation and should critically evaluate whether the construct definitions and intervention formats are ecologically appropriate for their target population.

The impact of measurement and design heterogeneity on synthesis precision warrants elaboration. At the measurement level, the construct of resilience is indexed by at least seven distinct validated instruments across the included studies, with pairwise inter-correlations ranging from *r* = 0.31 to *r* = 0.74; this range is wide enough that the lowest-correlated instruments may be measuring qualitatively different phenomena, rendering pooled effect size estimates for resilience interventions inherently imprecise even where study quality is high. Flow is similarly affected: the nine-item Flow Short Scale, the 36-item Dispositional Flow Scale-2, and the Experience Sampling Method do not reliably produce equivalent estimates of flow frequency or intensity (Moneta, 2012). At the design level, the mixing of RCTs, quasi-experimental designs, longitudinal observational studies, and cross-sectional designs within construct clusters means that observed heterogeneity in effect estimates reflects both true moderating variables (population, context, intervention format) and artefactual variance introduced by design differences in causal inference capacity. These sources of heterogeneity could not be disentangled in this narrative synthesis and represent a core reason why formal meta-analytic aggregation across construct clusters was not attempted.

Several steps were taken to reduce the subjectivity inherent in cluster classification. First, a pre-specified classification scheme was developed by the review team before data extraction, based on the theoretical taxonomy of positive psychology constructs outlined in Peterson and Seligman (2004) and updated by Seligman (2011); this scheme was documented in the registered protocol. Second, all borderline classification decisions defined operationally as cases where the primary construct examined by a study overlapped two cluster categories were referred to a three-way consensus discussion rather than resolved by a single reviewer. A total of 14 studies (16%) required this escalated consensus procedure. Third, inter-rater reliability for cluster classification was assessed using Cohen’s kappa across the 86 included studies prior to consensus resolution (*κ* = 0.79, 95% CI [0.72, 0.86]), indicating substantial agreement and suggesting that reviewer disagreement was concentrated in a predictable set of genuinely ambiguous cases rather than distributed randomly. Despite these precautions, residual subjectivity cannot be eliminated, and the five studies classified at the hope-optimism boundary and the three studies classified at the hedonic-eudaimonic boundary should be interpreted with awareness of their contested placement. Seventh, publication bias represents a persistent threat to the integrity of the synthesised effect size estimates that deserves explicit discussion in this Limitations section, in addition to the methodological account provided in Section 4.5. The grey literature exclusion decision (see Section 3.4) necessarily restricts the corpus to peer-reviewed publications, which are more likely to report statistically significant positive findings than null or negative results. Funnel plot asymmetry was detected in all 15 included meta-analyses, and trim-and-fill correction reduced pooled effect estimates by an average of 22% (range: 11–42%).

Eighth, paradigmatic incommensurability across included studies constitutes a structural limitation that the current synthesis cannot resolve. As discussed in Section 3.9, constructs such as resilience, well-being, and hope are operationalised from ontologically distinct theoretical positions—trait, process, and outcome paradigms for resilience; utilitarian hedonic and Aristotelian eudaimonic frameworks for well-being—whose underlying presuppositions are not mutually commensurable. Reporting similar effect sizes from studies with incommensurable paradigmatic foundations does not establish theoretical convergence; it establishes surface measurement similarity only. Future systematic reviews in this field should adopt paradigm-sensitive synthesis methods (e.g., Gough, 1987; Dixon-Woods et al., 2006), in which studies are first stratified by paradigmatic position and synthesised within paradigm before any cross-paradigm comparison is attempted, and in which interpretive conclusions are explicitly indexed to the paradigmatic assumptions of their contributing studies.

### 4.5 Conclusion

The journey from happiness to meaning tracks the intellectual maturation of positive psychology from a hedonic well-being science into a pluralistic framework of flourishing. Synthesising evidence from 71 primary studies and 15 meta-analyses, this review confirms genuine cumulative progress: gratitude (*d* = 0.31–0.38), resilience (*d* = 0.43), and character strengths (*d* = 0.29–0.45) are experimentally validated resources with replicable causal effects sustained after publication-bias correction. Meaning, self-determination, flow, and positive relationships show robust longitudinal associations with flourishing outcomes, though causal confirmation awaits further experimental work. The field’s central challenge remains construct proliferation: meeting it requires integrative theory, methodological standardisation, and sustained cross-cultural diversification.

This review makes four specific contributions. First, it provides the field’s first construct-level evidence hierarchy, distinguishing RCT-supported causal claims from observational associations. Second, it proposes and evidences the HPPIM—a four-tier integrative framework linking enabling resources through hedonic and eudaimonic well-being to authentic happiness and flourishing. Third, it advances methodological transparency through a two-metric effect size convention and trim-and-fill-adjusted estimates. Fourth, it provides the most comprehensive cross-cultural mapping of the evidence to date, documenting that 68% of studies originate from WEIRD contexts and benchmarking existing measurement invariance evidence for future validation work.

## Data Availability

The original contributions presented in the study are included in the article/Supplementary material, further inquiries can be directed to the corresponding author/s.

## Supplementary material

Supplementary Table S1 Exclusion rationale for constructs considered but not included in the systematic review (mindfulness, forgiveness, awe, and self-compassion), with full criteria applied against the four-point selection framework.

Supplementary Table S2 Complete data extraction form and coding scheme applied across all 86 included studies, including study design, sample characteristics, measurement instruments, effect sizes, and methodological quality ratings.

Supplementary Figure S1 PRISMA 2020 flow diagram illustrating the full study selection process from database search to final inclusion.
