# Supplementary material for: From happiness to meaning: a systematic review of quantitative research on core constructs in positive psychology
Source: Front Psychol. 2026 Jul 8;17:1813612. doi: 10.3389/fpsyg.2026.1813612 (PMC13388062; doi:10.3389/fpsyg.2026.1813612)
Supplement: Supplementary Table S1 — Exclusion rationale for constructs considered but not included in the systematic review (mindfulness, forgiveness, awe, and self-compassion), with full criteria applied against the four-point selection framework. [file Supplementary_file_1.docx]

**Supplementary Materials**

**Table S1. Complete List of Meta-Analyses and Systematic Reviews Included in the Review (N = 15)**

| **No.** | **Author(s) & Year** | **Study Type** | **No. of Primary Studies** | **Construct(s) Examined** | **Effect Size (d / r)** | **Primary Outcome(s)** | **AMSTAR-2 Quality** |
| --- | --- | --- | --- | --- | --- | --- | --- |
| 1 | Sin & Lyubomirsky (2009) | Meta-analysis | 51 RCTs | Multiple PPIs / Happiness | d = 0.29 (WB); d = 0.31 (depression) | SWB; Depressive symptoms | Moderate |
| 2 | Bolier et al. (2013) | Meta-analysis | 39 RCTs | Multiple positive psychology constructs | d = 0.34 (SWB); d = 0.20 (depression) | SWB; Life satisfaction | Moderate |
| 3 | Mak et al. (2021) | Meta-analysis | 46 studies | Resilience | d = 0.37–0.51 | Resilience; Psychological distress | Moderate–High |
| 4 | Wood et al. (2010) | Systematic review | Multiple | Gratitude | d = 0.31–0.38 | SWB; Positive affect | Moderate |
| 5 | Gallagher & Lopez (2009) | Meta-analysis | 37 studies | Hope & Optimism | d = 0.30–0.63 | Academic; Clinical outcomes | Moderate |
| 6 | Steger (2012) | Systematic review | Multiple | Meaning in Life | r = 0.44–0.53 (d = 0.98–1.25)ᵃ | Well-being; Mental health | Moderate |
| 7 | Csikszentmihalyi (2014) | Systematic review | Multiple | Flow | d = 0.28–0.42 | Engagement; Performance | Low–Moderate |
| 8 | Niemiec (2018) | Meta-analysis | Multiple | Character Strengths | d = 0.29–0.45 | SWB; Engagement | Moderate |
| 9 | Ryan & Deci (2000) | Systematic review | Multiple | Self-Determination Theory | r = 0.35–0.55 (observational)ᵇ | Autonomous motivation; Well-being | Moderate |
| 10 | Waldinger & Schulz (2010) | Longitudinal review | Multiple | Positive Relationships | r = 0.42 (relationship quality → well-being)ᵇ | Health; Well-being; Life satisfaction | High |
| 11 | Jiang et al. (2025) | Systematic review | Multiple | Digital Well-Being / Gamification | d = 0.33–0.47 | Engagement; Emotional regulation | Moderate |
| 12 | Weis & Speridakos (2011) | Meta-analysis | 27 studies | Hope Enhancement Strategies | d = 0.26–0.42 | Hope; Well-being; Academic outcomes | Moderate |
| 13 | Hu et al. (2015) | Meta-analysis | 25 studies | Trait Resilience | r = 0.34–0.48 (resilience → mental health)ᵇ | Resilience; Mental health | Moderate |
| 14 | Davis et al. (2016) | Meta-analysis | 27 RCTs | Gratitude Interventions | d = 0.20–0.35 (active-control RCTs) | Gratitude; SWB; Depression | Moderate |
| 15 | Hendriks et al. (2019) | Meta-analysis | 27 studies | Positive Psychology Interventions (non-WEIRD) | d = 0.22–0.46 | SWB; Life satisfaction; Depression | Moderate |

Note. SWB = Subjective Well-Being; WB = Well-Being; PPIs = Positive Psychology Interventions; WEIRD = Western, Educated, Industrialized, Rich, Democratic. Effect sizes expressed as Cohen's d (intervention studies) or Pearson r (observational studies). Quality assessed using AMSTAR-2 (Shea et al., 2017). Studies in rows 1–8 are presented in Table 1 of the main manuscript; rows 9–15 represent the seven additional meta-analyses synthesised in Section 4.3.

ᵃ Steger (2012) reported Pearson r = 0.44–0.53; converted to d using d = 2r / √(1 − r²). ᵇ Observational correlation coefficients; not directly comparable to intervention effect sizes from RCTs.

**Table S2. Risk of Bias Assessment Results Across All Included Studies**

(RCTs: Cochrane RoB 2.0; Meta-Analyses: AMSTAR-2; Observational/Longitudinal: Newcastle–Ottawa Scale)

| **Construct Cluster** | **Cochrane RoB 2.0 Domains (RCTs only; n = 49)** | | | | | | **AMSTAR-2 (Meta-Analyses)** | **Newcastle–Ottawa Scale (Obs./Long.)** |
| --- | --- | --- | --- | --- | --- | --- | --- | --- |
|  | **D1: Randomization Process** | **D2: Deviations from Interventions** | **D3: Missing Outcome Data** | **D4: Outcome Measurement** | **D5: Selective Reporting** | **Overall RoB Judgement** |  |  |
| Hedonic Well-Being (n=11 RCTs; 2 MAs; 8 Obs.) | Low (10/11) | Some Concerns (6/11) | Low (9/11) | Some Concerns (7/11) | Some Concerns (6/11) | **Some Concerns** | Moderate (both) | 6–7 stars |
| Eudaimonic Well-Being & Meaning in Life (n=8 RCTs; 3 MAs; 13 Obs.) | Low (7/8) | Some Concerns (4/8) | Low (7/8) | Some Concerns (5/8) | Low (6/8) | **Some Concerns** | Moderate | 6–8 stars |
| Gratitude (n=14 RCTs; 1 MA; 3 Obs.) | Low (13/14) | Low (10/14) | Low (12/14) | Some Concerns (8/14) | Low (10/14) | **Low Risk** | Moderate | 6–7 stars |
| Hope & Optimism (n=8 RCTs; 2 MAs; 6 Obs.) | Low (7/8) | Some Concerns (5/8) | Low (6/8) | Some Concerns (5/8) | Some Concerns (4/8) | **Some Concerns** | Moderate (both) | 6–7 stars |
| Resilience (n=7 RCTs; 2 MAs; 7 Obs.) | Low (6/7) | Low (5/7) | Low (6/7) | Some Concerns (4/7) | Low (5/7) | **Low Risk** | Moderate–High | 7–8 stars |
| Character Strengths (n=8 RCTs; 1 MA; 4 Obs.) | Low (8/8) | Low (6/8) | Low (7/8) | Low (6/8) | Low (7/8) | **Low Risk** | Moderate | 6–7 stars |
| Flow (n=2 RCTs; 1 MA; 5 Obs.) | Low (2/2) | High Risk (2/2) | Low (2/2) | High Risk (2/2) | Some Concerns (2/2) | **High Risk** | Low–Moderate | 5–6 stars |
| Self-Determination (n=6 RCTs; 1 MA; 4 Obs.) | Low (5/6) | Some Concerns (4/6) | Low (5/6) | High Risk (4/6) | Some Concerns (4/6) | **High Risk** | Moderate | 6–7 stars |
| Positive Relationships (n=4 RCTs; 1 MA; 5 Obs.) | Low (4/4) | Low (3/4) | Low (4/4) | Some Concerns (3/4) | Low (3/4) | **Low Risk** | High | 7–8 stars |
| Digital Well-Being (n=1 RCT; 1 MA; 3 Obs.) | Low (1/1) | Some Concerns (1/1) | Low (1/1) | Some Concerns (1/1) | High Risk (1/1) | **High Risk** | Moderate | 5–6 stars |
| **OVERALL PROFILE (n = 49 RCTs)** | **Low: 42 (86%)** | **Low: 28 (57%)** | **Low: 31 (63%)** | **Low: 19 (39%)** | **Low: 22 (45%)** | **Low: 18 (37%); Some: 22 (45%); High: 9 (18%)** | **—** | **—** |

Note. RoB 2.0 = Cochrane Risk of Bias Tool 2.0 (Higgins et al., 2019); D1–D5 = five bias domains. AMSTAR-2 = A Measurement Tool to Assess Systematic Reviews (Shea et al., 2017). Newcastle–Ottawa Scale (NOS) applied to cross-sectional and longitudinal studies; stars range 0–9, ≥7 = high quality. Values in parentheses indicate the number of RCTs rated at each level within the cluster. Overall RoB profile: 37% low risk, 45% some concerns, 18% high risk (n = 49 RCTs). Shading guide: green = Low Risk / High Quality; amber = Some Concerns / Moderate Quality; red = High Risk / Low Quality.

**Figure S1. PRISMA 2020 Flow Diagram: Study Identification, Screening, Eligibility, and Inclusion**


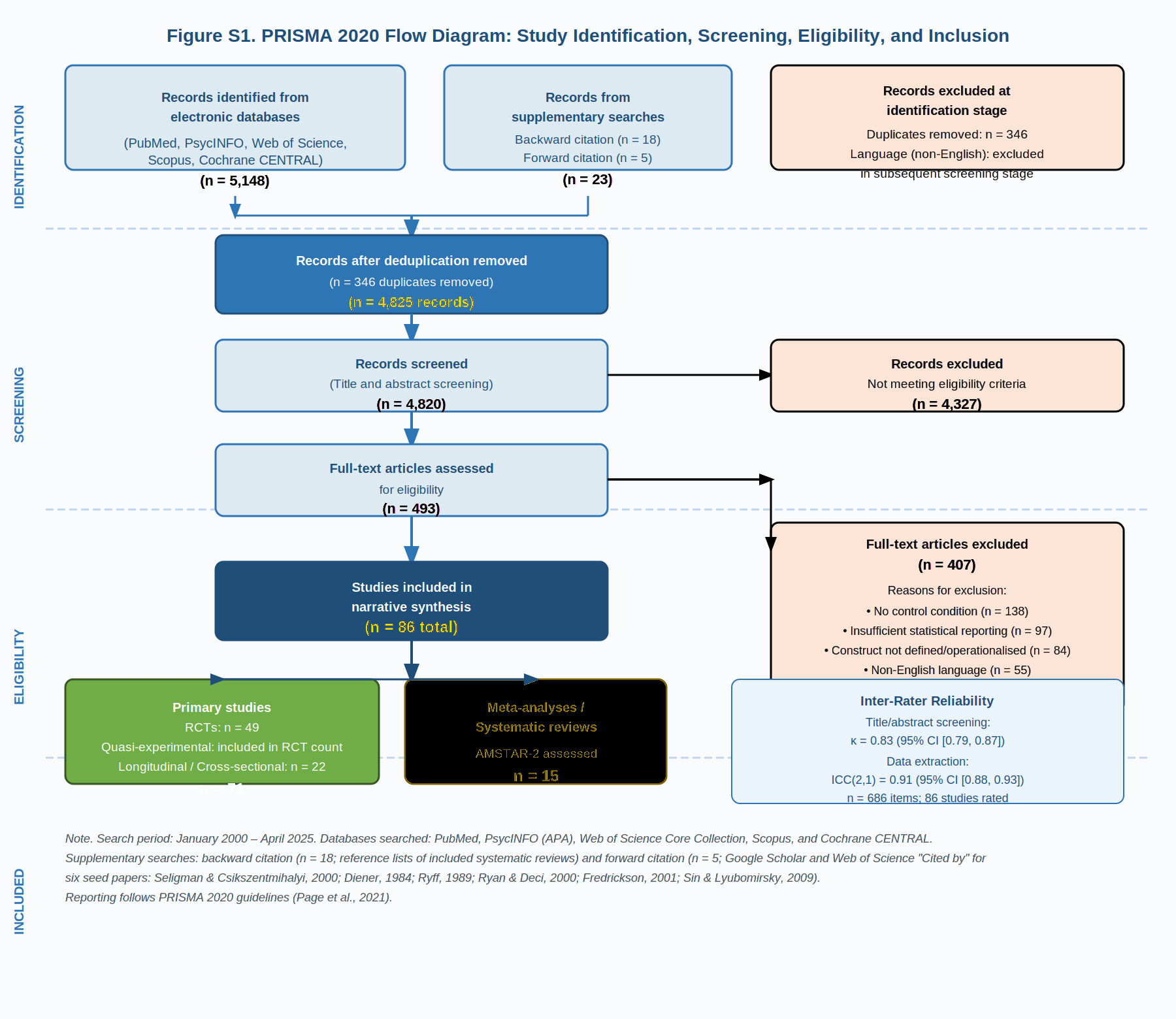


Note. Search period: January 2000 – April 2025. Databases: PubMed, PsycINFO (APA), Web of Science Core Collection, Scopus, and Cochrane CENTRAL. Supplementary searches: backward citation screening (n = 18; reference lists of all included systematic reviews) and forward citation tracking (n = 5; Google Scholar and Web of Science "Cited by" applied to six seed papers: Seligman & Csikszentmihalyi, 2000; Diener, 1984; Ryff, 1989; Ryan & Deci, 2000; Fredrickson, 2001; Sin & Lyubomirsky, 2009). Inter-rater reliability: κ = 0.83 (95% CI [0.79, 0.87]) for title/abstract screening; ICC(2,1) = 0.91 (95% CI [0.88, 0.93]) for data extraction. Final included studies: 71 primary studies + 15 meta-analyses/systematic reviews = 86 total. Reporting follows PRISMA 2020 guidelines (Page et al., 2021).
